# Primary Hyperparathyroidism: An Analysis Amid the Co-Occurrence of Type 2 Diabetes Mellitus

**DOI:** 10.3390/life15040677

**Published:** 2025-04-21

**Authors:** Ana-Maria Gheorghe, Mihaela Stanciu, Claudiu Nistor, Ioana Codruta Lebada, Mara Carsote

**Affiliations:** 1 PhD Doctoral School of “Carol Davila” University of Medicine and Pharmacy, 020021 Bucharest, Romania; ana-maria.gheorghe@drd.umfcd.ro; 2 Department of Endocrinology, Faculty of Medicine, “Lucian Blaga” University of Sibiu, 550024 Sibiu, Romania; codruta.lebada@ulbsibiu.ro; 3 Department of Endocrinology, Clinical County Emergency Hospital, 550245 Sibiu, Romania; 4 Department 4-Cardio-Thoracic Pathology, Thoracic Surgery II Discipline, “Carol Davila” University of Medicine and Pharmacy, 050474 Bucharest, Romania; 5 Thoracic Surgery Department, “Dr. Carol Davila” Central Military University Emergency Hospital, 010242 Bucharest, Romania; 6 Department of Endocrinology, “Carol Davila” University of Medicine and Pharmacy, 020021 Bucharest, Romania; carsote_m@hotmail.com; 7 Department of Clinical Endocrinology V, “C.I. Parhon” National Institute of Endocrinology, 011863 Bucharest, Romania

**Keywords:** primary hyperparathyroidism, diabetes, parathyroid, PTH, calcium, parathyroidectomy, glucose, insulin, hormone, surgery

## Abstract

Background: Apart from classical elements in primary hyperparathyroidism (PHPT), non-classical complications, including type 2 diabetes mellitus (T2DM), are reported in some patients, but currently, they do not represent a parathyroidectomy (PTx) indication. Objective: to explore the latest data regarding glucose profile, particularly, T2DM and metabolic syndrome (MetS) in PHPT, including post-PTx. Methods: PubMed-based review included English-published original studies between January 2020 and December 2024 (n = 20). Results: Studied population: 764,485 subjects (female-to-male ratio of 1.26:1; 23,931 were PHPT patients vs. 740,502 controls). T2DM prevalence (n = 13; N = 763,645 patients; 55.92% females): 4–60% (higher vs. controls); for the largest study (N = 699,157) of 31.3%. Age-based analysis: higher T2DM prevalence at >50 vs. <50 years (14.4% vs. 2.6%, *p* < 0.001), but not all studies agreed. Concurrent vitamin D deficiency as a contributor to a higher risk had limited evidence. The association MetS-PHPT (n = 2) had no clear conclusion. Post-PTx showed the following: lower glycaemia, fasting insulin, insulin resistance (HOMA-IR) improvement, and reduced rate (but not all studies agreed). PHPT patients with prediabetes might represent the population sub-group with the highest post-PTx benefit. Conclusions: The panel of PHPT-T2DM interplay remains heterogeneous. Data regarding post-PTx improvement of glucose disorders are still conflicting, recent findings suggested that surgery has beneficial effects, especially in patients with confirmed pre-existing prediabetes. Patients with the normocalcemic variant seemed to be less affected by the glucose-related disturbances, but further studies are needed. A better understanding of the intricate relationship between PHPT and glucose metabolism anomalies will help in providing optimal management to reduce the overall disease burden.

## 1. Introduction

Primary hyperparathyroidism (PHPT) represents a disease with a changing clinical picture in the modern era. While classical forms were often symptomatic, nowadays, asymptomatic and even normocalcemic and normohormonal presentations that are discovered during routine evaluations are increasingly more common [1,2,3]. The heterogeneity of the parathyroid tumour-related condition is also reflected in its complications and comorbidities: apart from the classical elements, such as osteoporosis and associated fragility fractures, nephrolithiasis, and even chronic kidney disease, a patient confirmed with PHPT might suffer from a wide spectrum of non-classical complications ranging from neuropsychiatric afflictions to cardiovascular and metabolic disturbances, including insulin resistance, prediabetes, and type 2 diabetes mellitus (T2DM) [4,5,6].

On the other hand, T2DM should be regarded as an ailment with a rising prevalence and high burden that might add to the one of PHPT [7,8]. An estimated 828 million adults were already affected by diabetes in 2022 [9], and projections suggested that up to 1.31 billion people might have the condition by 2050 [10]. A small population segment from these people might also suffer from PHPT and the relationship remains dual with many areas of uncertainty. The link between PHPT and T2DM, while it is still being refined, indicates towards a higher prevalence of T2DM in patients with PHPT that are seen in the general population, and a potential improvement in T2DM following parathyroid tumour removal has been reported. Some authors suggested that the increased intracellular calcium leads to insulin resistance by affecting insulin receptor activity and glucose transporters, but the connective mechanisms in subjects suffering from both PHPT and T2DM are multifactorial [11,12,13].

Nowadays, parathyroidectomy (PTx) is performed more often and at the early stages of PHPT with high cure rates of >90% and involves a small number of post-surgical complications when it is performed by an experienced surgeon [14,15,16]. While current guidelines clearly state the indication of PTx in patients with target-organ involvement, including osteoporosis and kidney stones, data regarding cardio-metabolic or neuro-psychiatric complications as an indication for surgery are insufficient; hence, a tailored decision is mandatory [17,18,19,20]. For instance, a recent meta-analysis suggested an improvement of glycaemic parameters following PTx, with a reduction in glucose levels of 0.16 [95% confidence interval (CI): −0.26, −0.06] mmol/L following PTx [21]. Furthermore, another study showed that patients with hypercalcaemic PHPT (HCPHPT) and normocalcemic PHPT (NCPHPT) had similar glucose parameters at baseline followed by a reduction in the insulin resistance after PTx in both groups [22]. However, other studies did not find a clear improvement in the glycaemic status following PTx [23]. A more recently recognized form of PHPT, NCPHPT, is usually associated with fewer complications; however, it usually benefits from PTx if associates a target organ involvement [24]. Whether glucose anomalies are more frequent in this distinct type of disease is still an open issue.

### Objective

The aim was to explore the latest data regarding glucose anomalies, particularly, T2DM and metabolic syndrome (MetS) in patients confirmed with PHPT, as well as the impact of PTx on glucose metabolism.

## 2. Methods

This is a PubMed-based, narrative review that included in the search only original English articles published between January 2020 and December 2024, using the following search keywords: “primary hyperparathyroidism”, “parathyroidectomy”, and “normocalcemic primary hyperparathyroidism” combined with “diabetes”, “prediabetes”, “glucose”, “insulin”, “metabolic syndrome”, and “HOMA”.

Original studies that provided data with respect to the glucose metabolism and associated disorders, including T2DM and prediabetes, as well as metabolic syndrome in individuals diagnosed with PHPT were included according to an open selection. We excluded case reports or case series, editorials, meta-analyses and systematic reviews, non-English papers, animal studies, and cohorts analysing hyperparathyroidism of secondary or tertiary type (including the parathyroid anomalies that are found in end-stage kidney disease), PHPT in pregnancy [25,26,27,28,29,30,31,32,33,34,35,36,37,38,39,40,41,42,43,44].

**Figure 1:**
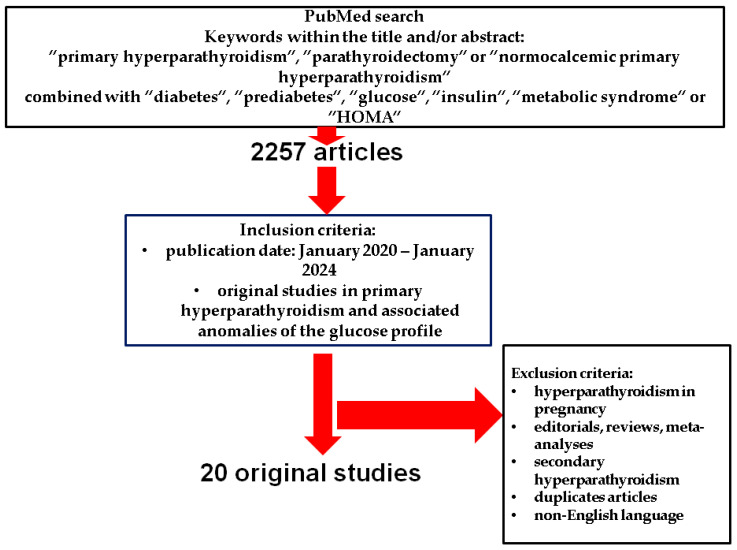
Flowchart strategy of search according to the above-mentioned methods [25,26,27,28,29,30,31,32,33,34,35,36,37,38,39,40,41,42,43,44,45].

Of note, we looked for glucose profile features in population sub-group diagnosed with PHPT, and did not include studied that searched for the diagnosis of PHPT in random diabetic population of any type (Figure 1).

## 3. Results

According to our methods, twenty studies reported data regarding the glucose metabolism and MetS in patients with PHPT, a total of 764,485 subjects [F(female):M(male) ratio of 1.26:1, meaning 55.93% were females), of whom 23,931 individuals were diagnosed with PHPT (F:M of 2.16:1; 68.41% females), and 740,502 subjects were PHPT-free (F:M of 1.24:1; 55.53% females) [25,26,27,28,29,30,31,32,33,34,35,36,37,38,39,40,41,42,43,44] (Table 1).

### 3.1. Sample-Focused Analysis

Five studies included a total of 145 patients with NCPHPT (F:M of 2:1, 33.79% females) [25,28,36,43,44]. The largest study population involved 699,157 individuals [32], while the largest population of patients with PHPT per study was of 11,616 [35]. Eights studies focused on prediabetes (and excluded T2DM) [28,30,36,38,40,41,43,44]. Of note, while some studies clearly defined T2DM based on ADA criteria [28,36,39,43,44,46], or WHO criteria [25,45], other studies (n = 5) relied solely on the prior electronic health records [26,32,33,35,37]. A 75 g oral glucose tolerance test (OGTT) was performed in three studies [41,43,44] and all of them reported data in patients without diabetes. Insulin resistance was confirmed based on HOMA-IR [28,36,40,43,44,47], HOMA2-IR [41], or the score recommended by the European Group for the Study of Insulin Resistance (EGIR) [28,48,49,50]. HOMA-B% [47] defined β-cell function [41,43,44]; HOMA-2S% [41] highlighted insulin sensitivity; additionally, QUICKI [41,50] and Matsuda Index [41,51] were also used to assess insulin sensitivity. MetS was defined based on the National Cholesterol Education Program Adult Treatment Panel III criteria [34,49]. Chen et al. [42] applied MetS score calculation based on waist circumference, high-density lipoprotein, triglycerides, fasting plasma glucose, and systolic blood pressure [52].

PHPT-related biochemical features included the following: mean/median serum calcium levels varied between 2.6 mmol/L [34] and 3 mmol/L [33] in PHPT (normocalcemic patients had average serum calcium between 2.4 mmol/L [25] and 2.47 mmol/L [44]). Control groups had mean calcaemic levels between 2.23 mmol/L [30] and 2.44 mmol/L [43]. Ionized calcium profile was provided in two studies [25,31], and average values were of 1.3 mmol/L in PHPT [31], respectively, of 1.2 mmol/L in NCPHPT, and 1.1 mmol/L in controls [25]. 24 h urinary calcium was reported by seven studies [25,26,27,31,34,38,39], with mean/median values varying between 3.3 mmol/24 h [26] and 11.3 mmol/24 h [27]. Mean (serum) phosphate in PHPT was between 0.75 mmol/L [27] and 0.92 mmol/L [40], while control groups had average levels between 1.1 mmol/L [29] and 1.19 mmol/L [25].

Subjects with PHPT had mean PTH values between 30 pg/mL [37] and 332.9 pg/mL [27] vs. controls patients with 29 pg/mL [25] and 60.16 pg/mL [35], and subjects with NCPHT—between 60 pg/mL [25] and 94.2 pg/mL [44].

Additionally, 25-hydroxyvitamin D (25OHD) levels in PHPT associated a mean 15.2 ng/mL [27], respectively, 25.7 ng/mL [35], respectively, while controls of 16 ng/mL [35] and 26.3 ng/mL [43]. Patients with NCPHPT had an average 25OHD of 31.2 ng/mL [43], respectively, of 38 ng/mL [25]. Compared to controls, patients with PHPT had similar 25OHD [20.8 (17.1–28.2) vs. 19.0 (13.3–21.9) ng/mL, *p* = 0.154 [30], and 40 (24–90) vs. 38 (24–63) nmol/L, *p* = 0.281 [35], respectively]. When patients with PHPT were divided in two groups based on 25OHD values (below and above 50 nmol/L), total albumin-adjusted calcium [2.6 (2.5–3.8) vs. 2.6 (2.6–3.4) mmol/L, *p* = 0.4], PTH [138 (65–700) vs. 135 (72–1229) ng/L, *p* = 0.8] and 24 h urinary calcium [311 (100–922) vs. 282 (98–1300) mg/24 h, *p* = 0.4] were similar [34].

To summarize, across twenty studies enrolling a very large population with PHPT, including normal-calcaemic and normal-hormonal sub-types, the prevalence/incidence and the spectrum of diabetes/pre-diabetes, insulin resistance (or even MetS to a lesser extend) were analysed starting from the individuals confirmed with the mentioned parathyroid condition. Of particular note, the female predominance which is already established in PHPT (that typically affects middle aged women in non-hereditary/syndromic forms) was confirmed in the overall studies population, as well [25,26,27,28,29,30,31,32,33,34,35,36,37,38,39,40,41,42,43,44].

### 3.2. Analysis of T2DM Prevalence/Incidence in Primary Hyperparathyroidism

T2DM prevalence or incidence (n = 13 [25,26,27,29,31,32,33,34,35,36,37,38,39]; N = 763,645 patients; F:M of 1.265:1; 55.92% were females) showed a prevalence between 4% [38] and 60% [26]. A total of 23,205 patients (F:M of 2.175:1; 68.52% females) had PHPT, and 740,440 subjects (F:M of 1.245:1; 55.53% females) were either controls or healthy subjects (without PHPT). The largest study, including 699,157 patients, out of which 6515 had PHPT, reported T2DM in 31.3% of PHPT patients [32]. The study with the most subjects diagnosed with PHPT (N = 11,616) confirmed T2DM in 8.1% of them [35]. PHPT patients compared to controls had a higher prevalence of T2DM: 31.3% vs. 9.3%, *p* < 0.001 [32]. Similarly, HCPHPT was associated with a higher prevalence of T2DM vs. controls and NCPHPT (35% vs. 12%, *p* < 0.05, and 35% vs. 12%, *p* < 0.05, respectively) [25].

However, a population-based study by Soto-Pedre et al. [35] reported a lower prevalence of T2DM in individuals with “probable” PHPT vs. controls (8.1% vs. 9.2%, *p* < 0.001). Yet, the study did not report the prevalence in those with definite PHPT diagnosis. On the other hand, the risk of T2DM was higher both in subjects with “probable” PHPT [HR (95% CI) = 1.39 (1.26–1.54), *p* < 0.05] and “definite” PHPT [HR (95% CI) = 1.43 (1.28–1.60), *p* < 0.05] [35].

Additionally, the age-based analysis showed a higher prevalence of T2DM in patients older than 50 y vs. younger than 50 y (14.4% vs. 2.6%, *p* < 0.001) [31]. Similarly, a retrospective study on 130 females found a higher prevalence of T2DM in menopause compare to pre-menopause (16.7% vs. 3.5%, *p* = 0.033) [33].

Vitamin D status might influence T2DM prevalence, while not all studies agree, for instance, a retrospective study reported a similar prevalence of T2DM in subjects with PHPT and 25-OHD < 50 nmol/L compared with PHPT and 25-OHD ≥ 50 nmol/L [34]. When adjusted for the vitamin D level, the risk of T2DM was higher in subjects with PHPT patients vs. healthy controls [HR (95% CI) = 1.26 (1.07–1.48), *p* < 0.05) [35]. Two studies provided the prevalence of T2DM (of 13.19% [36], and of 4.01% [38]), despite T2DM was an exclusion criteria for the final analysis.

A large retrospective population-based cohort study on 16,494 subjects (2749 with PHPT and 13,745 matched controls) reported an incidence rate (95% CI) of T2DM of 27.6 (25.00–30.00) per 1000 person-year in PHPT and 23.90 (22.80–24.90) per 1000 person-year in controls, with a 15% higher overall risk in PHPT [HR (95% CI) = 1.15 (1.04–1.28), *p* = 0.007]. The risk remained statistically significant after adjustment for screening frequency [HR (95% CI) = 1.12 (1.01–1.24)]. PHPT patients and serum calcium above the median (2.63 mmol/L) had higher incidence rate compared to those with lower serum calcium levels (28.80 vs. 26.50 per 1000 person-year, *p* = 0.001) and a 44% higher risk of T2DM [HR (95% CI) = 1.44 (1.08–1.90)] [29]. Of interesting note, other collateral findings included a higher rate of pancreatitis and nephrolithiasis in subjects with PHPT and T2DM vs. PHPT without T2DM [39].

To conclude, according to 13 studies, the prevalence of T2DM in patients confirmed with PHPT was heterogonous, from 4% to 60%, and seemed higher vs. controls and increased over 50 y or in post-menopause. Whether the concurrent anomalies of vitamin D profile might contribute to a higher risk of T2DM in PHPT vs. controls associated a limited statistical evidence [25,26,27,29,31,32,33,34,35,36,37,38,39] (Table 2).

### 3.3. Prediabetes and Insulin Resistance in Patients with Primary Hyperparathyroidism

The prevalence of prediabetes and/or insulin resistance was reported by four studies, including 1308 patients with PHPT (F:M of 6.67:1; 86.7% females) [28,31,36,40]. Notably, three of these studies (N = 470; F:M of 3.35:1; 76.38% females) only included diabetes-free patients with PHPT [28,36,40]. The prevalence of prediabetes, defined either as impaired fasting glycaemia (IFG) and/or impaired glucose tolerance (IGT), was analysed in three studies (N = 1243; F:M of 7.66:1; 88.17% females) [28,31,36]. ADA [46] criteria were used to assess IFG (n = 2) [28,36], while the third study did not specify IGF/IGT criteria [31].

The prevalence of prediabetes varied between 2% [31] and 36% [36]. Al-Jehani et al. [36] found it to be higher in PHPT vs. controls (36% vs. 26%, *p* = 0.035). This rate was similar across different types of PHPT (NCPHPT vs. mild HCPHPT vs. classic HCPHPT: 23% vs. 39% vs. 41%, *p* = 0.176) [36]. Dobreva et al. [31] compared the prediabetes prevalence between distinct age groups (18–49 y vs. ≥50 y) and reported that, although younger patients had a lower prevalence, the statistical significance was lost after Bonferroni correction (2% vs. 7.3%, *p* = 0.016) [31]. The influence of PTx was analysed by Nomine-Criqui et al. [28] who reported a similar prevalence of prediabetes before, and 3 months, 6 months and 1 year following PTx (32% vs. 39% vs. 35% vs. 35%, *p* = 0.555) [28].

Three studies (N = 470; F:M of 3.35:1; 76.38% females) analysed the prevalence of insulin resistance via HOMA-IR with different thresholds (≥2.5 [28,40], respectively, >2.6 [36]) or via EGIR criteria (HOMA-IR > 1.8) [28,36,40]. Based on HOMA-IR, insulin resistance affected between 32.3% [40] and 47% [28] of PHPT patients. NCPHPT associated a lower insulin resistance rate than HCPHPT (either mild or classic form): 17% vs. 43% vs. 70% (*p* < 0.001). Subjects with PHPT had a higher prevalence of insulin resistance vs. controls (20% vs. 45%, *p* < 0.001) [36].

PTx had a beneficial effect on reducing the rate of insulin resistance (32.3% vs. 23.1%, *p* = 0.031) in one study [40]. However, Nomine-Criqui et al. [28] did not confirm a statistically significant difference following PTx (47% vs. 50% vs. 47% vs. 50%, *p* = 0.514) [28].

Overall, IFG/IGT was addressed across a smaller number of studies (n = 4) than T2DM (n = 13), and showed different rates in PHPT (from 2% up to 36%), a prevalence that was higher than controls and displayed no particular age-related pattern. On the other hand, insulin resistance (confirmed via slightly different cut offs in 32% to 76% of the subjects with parathyroid tumours) seemed more frequent than found in control groups. The statistical evidence to highlighting that IFG/IGT or insulin resistance improved after parathyroid tumour removal remains scarce [28,31,36,40] (Table 3).

### 3.4. Glucose Profile Assays (Fasting Glycaemia, Insulin and HBA1c) in Primary Hyperparathyroidism

Twelve studies [N = 2412 subjects; F:M of 4.82:1; 82.83% females, of whom 2093 were confirmed with PHPT (F:M of 5.27:1; 87.42% females)] showed that in PHPT patients mean fasting glucose varied between 87.55 mg/dL [40] and 5.44 mmol/L (98 mg/dL) [28,36]; the highest mean values were of 6.272 mmol/L (113 mg/dL) in HCPHPT [25]. Two studies focused on patients with NCPHPT and prediabetes, and mean fasting glucose was of 5.86 mmol/L (105.6 mg/dL), respectively, of 6.62 mmol/L (119.4 mg/dL) [43,44].

Subjects with PHPT vs. controls associated either similar fasting glucose levels or higher in PHPT across different studies. Specifically, a comparative study reported similar median (IQR) fasting glucose between patients with PHPT and controls [5.04 (4.63–5.23) vs. 4.83 (4.5–5.2) mmol/L, *p* = 0.556] [30], while another identified a higher fasting glucose in PHPT vs. controls (0.98 ± 0.13 vs. 0.93 ± 0.12 mmol/L, *p* < 0.001). A potential bias was the older age in PHPT vs. controls (61.8 ± 14.6 vs. 37.3 ± 8.2 y, *p* < 0.05) [36]. Dobreva et al. [31] reported lower mean (IQR) fasting glucose levels in patients with PHPT without T2DM (vs. diabetics), and younger than 50 y (vs. older than 50 y) [31].

Mean/median HbA1c varied between 5% [40] and 5.4% [26] in PHPT. Subjects with NCPHPT and prediabetes had mean HbA1c between 5.84% and 5.9% [43,44]; those with PHPT and T2DM had an average HbA1c of 7.2% [39]. Older individuals with PHPT had higher HbA1c levels compared to younger patients [5.6 (5.3–5.8) vs. 5.3 (5.0–5.4), *p* = 0.002] [31].

Mean fasting insulin varied between 8.7 mUI/L [41] and 13.6 mUI/L [30]. In patients with NCPHPT and prediabetes, mean insulin levels were between 11 µIU/mL [44] and 14 µIU/mL [43]. Subjects with PHPT had higher fasting insulin vs. controls (13.6 ± 12.3 vs. 8.07 ± 4.16 mUI/L, *p* < 0.001), especially in subjects with classic HCPHPT, who showed increased levels compared to mild HCPHPT and NCPHPT (17.73 ± 12 vs. 13.34 ± 13.76 vs. 8.92 ± 5 mUI/L, *p* = 0.005) [36]. Bibik et al. [30] found an elevated insulin AUC (aria under curve) in PHPT patients vs. controls [61.9 (44.4–73.9) vs. 37.6 (36.1–42.6), *p* < 0.001] [30] (Table 4).

### 3.5. 75-g Oral Glucose Tolerance Testing in Individuals with Primary Hyperparathyroidism

75-g OGTT in PHPT was performed in three studies [41,43,44]. A total of 66 patients diagnosed with PHPT (F:M of 2.88:1; 74.24% females), including 52 of them who were confirmed with NCPHPT (F:M of 2.46:1; 71.15% females), and 42 controls (F:M of 2.5:1, 71.43% females). Post-surgery analysis was provided in 30 subjects [41,43,44].

In PHPT, OGTT response showed similar before and after surgery values of glucose, insulin and gastric inhibitory polypeptide (GIP) levels. However, glucagon-like peptide 1 (GLP-1) levels were higher following parathyroid surgery both at 60 min (63.06 ± 44.78 vs. 102.64 ± 40.19 pg/mL, *p* = 0.02) and 120 min (71.20 ± 35.9 vs. 102.49 ± 40.02 pg/mL, *p* = 0.03) during the OGTT [41].

OGTT in NCPHPT individuals who were surgically vs. conservatively managed showed that both groups had a similar 2-h post-load glucose levels at baseline (163.2 ± 3.2 vs. 167.2 ± 3.2 mg/dL, *p* = 0.371). After PTx in the first group and conservative follow-up of 32 weeks in the second group, prediabetic patients who underwent PTx had lower 2-h post-load glucose levels compared to those who were conservatively managed (144.2 ± 3.2 vs. 176.2 ± 3.2 (−32 ± 0.4) mg/dL, *p* < 0.01). Moreover, glucose levels during OGTT were lower after PTx in surgery group (163.2 ± 3.2 vs. 144.4 ± 3.2 (−18.8 ± 0.3) mg/dL, *p* = 0.041) [44]. Another study pinpointed patients with PHPT and prediabetes who showed a similar 2-h post-load glucose level during OGTT compared to a control (non-PHPT) population with prediabetes (157.2 ± 2.2 vs. 152.2 ± 2 mg/dL, *p* = 0.07) [43] (Table 5).

### 3.6. Primary Hyperparathyroidism: Specific Considerations in the Field of Insulin Resistance and Insulin Sensitivity

Insulin resistance in patients with PHPT was analysed across eight studies. Two of them included strictly NCPHPT patients [a total of 956 subjects (F:M of 2.57; 76.98% females), out of whom 709 individuals (F:M of 3.43; 77.43% females) had PHPT and 247 controls (F:M of 2.36; 75.7% females), respectively, 104 patients were confirmed with NCPHPT (F:M of 1.15; 47.11% females)]. Insulin resistance was assessed by HOMA-IR (n = 8), while β-cell function was evaluated by HOMA-B% (n = 6). Insulin sensitivity was analysed using HOMA-S% (n = 1), QUICKI (n = 1), and Matsuda index (n = 1) [25,28,33,36,38,40,41,43].

The highest mean HOMA-IR was of 3.39 [36], while the lowest was of 1.14 [41]. The highest β-cell function (HOMA-B%) was 156 ± 262 [36] vs. the lowest of 97.53 ± 25.13% [41]. The largest study investigating insulin resistance included 174 PHPT patients (vs. 171 controls), and they had an increased insulin resistance (HOMA-IR: 3.39 ± 3.11 vs. 1.92 ± 1.16, *p* < 0.001) [36]. This cohort also provided data regarding the potential influence of disease severity, comparing NCPHPT, mild HCPHPT and classic PHPT: the lowest insulin resistance was found in normocalcemic variant vs. mild HCPHPT and classic HCPHPT (HOMA-IR: 2.14 ± 1.29 vs. 3.28 ± 3.29 vs. 4.53 ± 3.51, *p* = 0.002), but with similar β-cell function (HOMA-B%: 105.9 ± 57.4 vs. 165.9 ± 348.7 vs. 176.4 ± 107.7, *p* = 0.447) [36].

Another study on 231 PHPT patients analysed postoperative insulin resistance in prediabetic vs. non-prediabetic patients at baseline and found lower values after PTx in subjects with pre-existing prediabetes (4.79 ± 3.49 vs. 4.1 ± 2.38, *p* = 0.040), and no difference in the prediabetes-free subjects, suggesting a post-surgery improvement of the insulin resistance if the baseline level qualifies for IFG/IGT [28]. Patients with NCPHPT and prediabetes who were either treated surgically or conservatively had a similar HOMA-IR and HOMA-B%, as shown by Karras et al. [44]. Also, Barale et al. [25] identified a similar HOMA-IR between NCPHPT and HCPHPT (HOMA-IR: 1.1 ± 0.5 vs. 2.7 ± 1.5, *p* > 0.05) [25]. Additionally, post-surgery HOMA-IR decreased in two cohorts: 2.3 ± 1.6 vs. 1.9 ± 1.3, *p* < 0.001 [38], and 2.21 ± 0.69 vs. 2.02 ± 0.64, *p* = 0.0001 [40], and remained stationary in another study (3.29 ± 2.79 vs. 3.07 ± 2.13, *p* = 0.514) [28]. One pilot study of a small sample size (N = 14) investigated insulin sensitivity and reported a mean HOMA-S% of 127.74 ± 76.90% with a post-operatory value of 104.51 ± 48.15% that was not statistically significant (*p* = 0.68). Neither QUICKI, nor Matsuda index found differences following parathyroid surgery (QUICKI: 0.36 ± 0.04 vs. 0.34 ± 0.03, *p* = 0.08 and Matsuda index: 6.34 ± 3.7 vs. 5.27 ± 2.44, *p* = 0.06) [41] (Table 6).

### 3.7. Identifying Metabolic Syndrome in Individuals with Primary Hyperparathyroidism

Two studies provided data regarding MetS in PHPT [520 individuals (F:M of 1.68:1; 62.7% females)] [34,42]. In a retrospective study on 128 PHPT subjects (F:M of 4.8:1; 82.8% females), MetS was analysed in relation with 25OHD. Although patients with MetS vs. (MetS-free) controls did not have a statistically significant difference in terms of median (IQR) 25OHD [40.8 (10–150.8) vs. 52.8 (10–206.8), *p* = 0.52], subjects with vitamin D deficiency (25OHD < 50 nmol/L) had a higher prevalence of MetS compared to the sub-group with sufficient vitamin D levels (40.9% vs. 24.2%, *p* = 0.04) [34]. Another study, on 392 subjects with elevated PTH levels (F:M of 1.28:1; 56.21% females) found a positive correlation between MetS score and PTH in the entire group (β = 0.399, *p* = 0.030), and in distinct subgroups such as those with moderate physical activity (β = 0.413, *p* = 0.045), without vitamin D supplementation (β = 0.524, *p* = 0.028), without vitamin D deficiency (β = 0.456, *p* = 0.014), and with high protein intake (β = 0.586, *p* = 0.03) [42] (Table 7).

### 3.8. Impact of Parathyroidectomy on Glucose Metabolism

Data about the outcome of PTx was provided by seven studies, with a total of 599 patients (F:M of 3.87:1; 79.46% females), of whom 16 NCPHPT subjects (F:M of 3:1; 75% females) [26,28,30,38,40,41,44]. Most data regarding the impact of PTx on glucose metabolism supported the fact that fasting plasma glucose and insulin resistance reduced after surgery [28,30,38,40]. However, some studies did not support these results [41].

The largest study (of observational design) in 231 PHPT patients who underwent surgery, showed a similar fasting plasma glucose and HOMA-IR before and 3 months, 6 months and one year after the procedure. However, a separate analysis of a subgroup of patients with prediabetes revealed a reduction in HOMA-IR, as mentioned. Similarly, HOMA-IR decreased upon PTx in patients with preoperative insulin resistance and in those with insulin resistance according to EGIR criteria. The change in HOMA-IR negatively correlated with serum calcium (r = −0.173, *p* = 0.008) [28]. Another observational prospective study on 139 PHPT patients who underwent PTx showed a post-surgery improvement of the glucose metabolism parameters such as fasting plasma glucose and insulin, while the change in the β-cell function was not statistically significant.

Moreover, following PTx, adiponectine levels were higher (6.2 ± 3.6 vs. 7.2 ± 3.9 μg/mL, *p* < 0.001). The change on plasma glucose, fasting insulin, HOMA-IR, HOMA-B%, and adiponectin did not correlate with the change in calcium or PTH as corrected upon parathyroid tumour removal [38]. A retrospective cohort study in 65 surgery candidates with PHPT showed lower fasting plasma glucose, insulin, HbA1c, HOMA-IR, and a reduction the insulin resistance prevalence (32.3% vs. 23.1%, *p* = 0.031) [40]. Bibik et al. [25] reported decreased glycaemia [5.10 (4.81–5.24) vs. 4.69 (4.48–5.00) mmol/L, *p* = 0.031], but increased HbA1c [5.30 (5.10–5.50) vs. 5.60 (5.30–5.80) %, *p* = 0.001] after PTx. Insulin resistance, as assessed by the glucose uptake at tissues (M-value) was similar before and after surgery [5.48 (4.30–7.43) vs. 6.17 (4.56–6.90) mg/kg/min, *p* = 0.959] [25]. Interestingly, Govind et al. [26] found higher HbA1c levels in subjects who post-operatory developed hungry bone syndrome after PTx [5.4 (5.3–5.8) vs. 6.3 (5.8–7.9) %, *p* = 0.008] [26] (Appendix A).

## 4. Discussion

In this large sample-based analysis, we identified various rates of T2DM in PHPT, highly suggestive for an increased prevalence vs. general population with lower levels of evidence with concern to IFG/IGT and a potential post-surgical improvement of the abnormal glucose and metabolic features following the removal of the underlying parathyroid tumour. Yet, the spectrum remains heterogeneous from a statistical perspective and these types of analyses are needed to raise concerns regarding T2DM in these patients and to increase awareness with additional elements that might contribute to disease burden other than traditional complications of long term high calcium and PTH [25,26,27,28,29,30,31,32,33,34,35,36,37,38,39,40,41,42,43,44].

### 4.1. The Interplay Between PTH and the Metabolic Profile, Including Hormonal Glucose Regulation

As mentioned, the landscape of the non-PTH hormonal interplay in the metabolic regulation and PHPT was assessed in three studies [30,38,41] according to our sample-focused analysis: adiponectin (n = 2, N = 183, including 163 patients with PHPT and 20 controls) [30,38], leptin (n = 1, N = 24 with PHPT and 20 controls) [30], GIP and GLP-1 (n = 1, N = 14 patients with PHPT who underwent PTx) [41]. Adiponectin was found similar between subjects with PHPT vs. controls [30], but increased following parathyroid surgery in individuals with classical and mild of parathyroid condition [38], hence, suggesting a potential connection to the PTH changes, but further evidence is needed. Leptin was similar between patients with PHPT and controls, as well as pre- and post-operatory [30]. Antonopoulou et al. [41] investigated GIP and GLP-1 levels in 14 subjects with PHPT who underwent PTx. There was no statistically significant change neither for GLP-1 levels (74.73 ± 52.33 vs. 59.25 ± 25.67 pg/mL, *p* = 0.58), neither for GIP (3.45 (7.43) vs. 9.84 (29.59) pg/mL, *p* = 0.26) [41] (Table A2).

Overall, despite the clinical evidence that has been mentioned, no clear relationship had been established between the levels of PTH and the hormones that are active in the glucose regulation. Generally, apart from the classical effects of bone remodelling and regulation of the calcium homeostasis, PTH has been linked to various metabolic effects, especially regarding energy metabolism [53,54,55,56]. On lipid metabolism, PTH promotes lipolysis by activating the hormone sensitive lipase, as showed by in vitro studies [57]. According to some clinical evidence, PTH positively correlates with body fat mass as found in healthy females [58] and males with chronic kidney disease on dialysis [59]. Moreover, in vitro human studies and animal models have shown that PTH induces thermogenic effects in adipocytes contributing to the adipocyte browning effect [60,61,62,63].

Another role of PTH in osteoblasts is the activation of energy production both by stimulating glycolysis and by enhancing the mitochondrial electron transport chain function [64,65]. However, clear evidence regarding molecular pathways induced by prolonged PTH secretion is lacking and is therefore difficult to assess the mechanisms beneath PTH effects on glucose and lipid metabolism in patients with PHPT [56].

Prior data have shown the involvement of PTH in insulin resistance development. In vivo studies found that PTH has an inhibitory effect on insulin signalling in differentiated adipocytes, decreasing glucose uptake upon the stimulation of adipocytes by insulin, and reducing glucose transporter type 4 and insulin receptor substrate-1 expression [66]. Similarly, murine models revealed that PTH infusion and consequent hypercalcemia were associated with higher glucose levels and similar insulin levels, while both PTH and calcium positively correlated with glucose levels only in rats who received PTH injections [67]. Regarding physiological PTH levels, one recent study showed in patients with a novel T2DM a negative association between PTH levels and HbA1c at diagnosis of T2DM (β = −1.475, *p* = 0.003), and a positive association with HOMA-B% (β = 0.090, *p* = 0.001) [68].

On the other hand, PTH-induced hypercalcemia might be a contributor to the glucose status disturbances under certain circumstances, including the co-presence of obesity or other non-diabetes metabolic anomalies [69,70,71]. Increased intracellular calcium in skeletal muscles stimulates phosphorylation of the glycogen phosphorylase, an enzyme that converts glycogen to glucose. At the same time, high intracellular calcium inhibits glycogen synthetase through the activation of phosphokinase. Moreover, it was linked to reduced glucose transporter type 4. These processes result in glycogenolysis and hyperglycaemia contributing to the development of T2DM [69]. Calcium signalling is also a key factor in insulin secretion by β-cells. For instance, intracellular calcium is transported inside the mitochondrial matrix by mitochondrial Ca^2+^ uniporter. Once inside the mitochondria, Ca^2+^ then modulates β-cell signal transduction, activating glucose-induced insulin secretion [71,72,73]. Of note, another entity that may be involved in the development of diabetes in patients with PHPT is hypercalcemia-related pancreatitis. Whether acute or chronic, it is a relatively rare occurrence. While the risk is still being debated, it may sometimes be the first manifestation of the parathyroid condition [74,75,76].

Under pathological circumstances of a parathyroid tumour-related hypercalcemia, as found in PHPT, another possible bridge between PHPT and glucose metabolism is via bone status, considering the bidirectional relationship between the bone metabolism and glucose metabolism [77,78,79]. Osteoblasts have an active metabolism and among the molecules secreted by them, osteocalcin and lipocalin-2 also have regulatory roles on basal energy metabolism [79]. Data from animal studies have shown that osteocalcin stimulates insulin secretion, as mice lacking osteocalcin developed reduced β-cell proliferation, glucose intolerance and insulin resistance [80,81]. Apart from stimulating pancreatic β-cells, osteocalcin also influences testosterone and GLP-1 secretion [82]. On the other hand, insulin exerts an anabolic effect on bone, by stimulating osteoblast proliferation, collagen synthesis and glucose uptake, at physiological levels [83]. In addition, data from animal models indicate that insulin resistance in bone negatively impacts osteocalcin, and further impairs the glucose metabolism, when the animals were fed a high fat diet [84].

**Figure 2:**
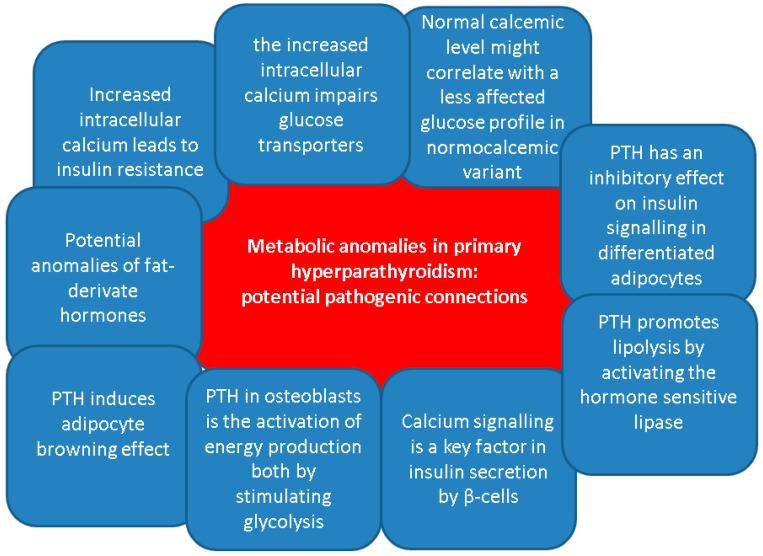
Potential pathogenic connections between primary hyperparathyroidism and metabolic anomalies, including glucose metabolism [71,72,73,74,75,76,77,78,79,80,81,82,83,84,85,86,87,88,89,90,91,92,93].

Additionally, diabetic bone disease and high PTH-induced osteoporosis might inter-connect in the same patient, while sarcopenia, as a complication of both ailments, represents a supplementary osteoporotic fracture risk (due to an increased risk of fall) [85,86,87]. No particular study amid our search highlighted the osteoporosis/osteopenia status in subjects who display both T2DM and PHPT. Notably 25% to 40% of the T2DM population might suffer from “sweet bones”, particularly, in certain population sub-groups such as menopausal females [88,89,90]. Of interesting note, the highest prevalence of non-hereditary PHPT is also in women within their fifth and sixth decade of life and this might bring a potential bias in the interpretation of the bone involvement amid the co-diagnosis of in PHPT with T2DM [91,92,93] (Figure 2).

### 4.2. Should Parathyroidectomy Be Indicated Based on the Presence of T2DM?

PTx is the curative treatment of PHPT and current guidelines clearly state the criteria based on bone and kidney involvement or high blood calcium levels, but not based on the presence of T2DM or MetS. However, PTx may also be performed in the absence of these specific (guideline-based) criteria as long as the patient does not have contraindications across a multidisciplinary patient-tailored decision depending on the particular aspects of each case. The evidence regarding the beneficial effect on glucose metabolism is considered insufficient to include glucose disorders among PTx indications as far as we know at this point [94,95,96].

As mentioned, recent studies have explored the effects of PTx on glucose metabolism, and overall, a potential benefit of PTx on glucose status is noted: e.g., lower fasting plasma glucose [30,38,40], decreased fasting insulin [38,40] and improvement of the insulin resistance as assessed by HOMA-IR [38,40] as well as a reduction of insulin resistance prevalence following parathyroid surgery [40]. However, other authors did not confirm a statistically significant difference before and after PTx with respect to the fasting plasma glucose [28,41], fasting insulin levels [28,41], insulin resistance and OGTT [41].

The population sub-group that might particularly benefit from surgery includes patients with PHPT and prediabetes, as already specified [28]. Furthermore, in subjects confirmed with NCPHPT and prediabetes who underwent surgery fasting plasma glucose and 2-h post-load glucose were lower postoperatively compared with patients with NCPHPT who were managed conservatively for 32 weeks [44]. Another interesting finding is represented by a decreased HbA1c in patients who developed hungry bone syndrome after surgery [26]. This is a post-PTx entity caused by the fast reduction of PTH levels with subsequent bone remineralization leading to severe hypocalcaemia [97,98,99]. One possible hypothesis connecting the levels of HbA1c to the hungry bone syndrome involves a reduced bone turnover at higher HbA1c values in patients with T2DM [100,101,102,103]. Further research is needed in the matter of this distinct association.

### 4.3. Glucose-Related Insights into Novel Forms of PHPT (Normocalcemic Variant)

According to our methods, four studies included a total of 104 patients with NCPHPT, 198 subjects with HCPHPT and 76 controls [25,36,43,44] with overall discordant findings regarding glucose profile impact. For instance, the relationship between the severity of the parathyroid condition and the glucose metabolism was explored by Al-Jehani et al. [36] in a small-sized study and, while glycaemic status was similar between the groups, NCPHPT patients had a better insulin resistance profile than classical PHPT [36]. Two studies analysed patients with NCPHPT and prediabetes: fasting plasma glucose was higher in patients with NCPHPT and prediabetes vs. controls with prediabetes, but similar HbA1c, fasting insulin levels, HOMA-IR, HOMA-B%, as well as OGTT [43]. Following PTx, however, patients with NCPHPT showed an improvement of the fasting plasma glucose and OGTT response [44]. Barale et al. [25] found a similar prevalence of T2DM, fasting plasma glucose, fasting insulin and insulin resistance between NCPHPT and controls. Furthermore, the authors found a lower prevalence of T2DM, and glucose disorders, and decreased fasting plasma glucose in patients with NCPHPT compared to HCPHPT [25] (Table A3).

Notably, NCPHPT has been recognized as a distinct form of PHPT during latest years due to an early detection via various biochemistry screening protocols in general population, but there are no specific guidelines regarding surgery. While the condition has been linked to the similar comorbidities and complications as HCPHPT, such as osteoporosis and urolithiasis, currently, a standardized treatment approach is still missing. In addition, more controversial complications such as glucose disorders are not fully understood and placed in relationship with the interventional management [103,104].

### 4.4. Limits and Further Research

As limits of the current analysis we mention the non-systematic design which was preferred in order to expand the area of included studies, hence, providing a larger area of various assays with respect to the mineral and glucose metabolisms in PHPT. As potential bias we should mention the different designs of the analysed studies, mostly of retrospective type. Notably, some of them included a small sample size (of less than 100 patients) which limited the statistical evidence and impact the final results. As mentioned, no specific data in the paediatric population or hereditary PHPT were available. Further expansion in the cross-field of T2DM-PHPT interplay is mandatory, not only with respect to the glucose profile assessments and management, but, also, with regard to other common complications, including osteoporosis and fragility fractures as well as kidney damage. Whether particular genetic or epigenetic contributors increase the diabetic risk in a sub-category of adults diagnosed with PHPT is still an open mater. As mentioned, T2DM does not represent (yet) a stand-alone indication for parathyroid surgery, but, amid its multimodal management, the presence of T2DM in addition to other high calcium- and high PTH-related complications might help the decision of PTx. Additionally, we found no data with regard to a specific anti-diabetic/interventional approach in T2DM-PHPT and further studies might be useful.

## 5. Conclusions

Based on the sample-focused analysis in more than 700,000 subjects, the rate of T2DM in PHPT might include two thirds of the patients in some studies, but the frequency varies. IGT/IFG is less often described than T2DM, but the panel of results remains heterogeneous. While data regarding PTx in patients with PHPT and glucose disorders are still conflicting, recent findings suggested that PTx has beneficial effects regarding insulin resistance and fasting plasma glucose, especially in patients with confirmed pre-existing prediabetes. Patients with normocalcemic variant seem to be less affected by the glucose disorders, but further studies are needed. In order to provide a personalised management, future research goals should include a key-finding strategy to identify the patients who would mostly benefit from T2DM screening in PHPT or from parathyroid surgery once the presence of T2DM has been established. Another area of interest is represented by the interconnection and possible cumulative effects on comorbidities such as osteoporosis and fracture risk between PHPT and T2DM, but current data are scarce. A better understanding of the intricate relationship between glucose metabolism anomalies and PHPT will help providing an optimum management in order to reducing the overall diseases’ burden.

## Figures and Tables

**Table 1:** Included studies in the focused-sample analysis according to our methods [25,26,27,28,29,30,31,32,33,34,35,36,37,38,39,40,41,42,43,44].

First Author Year of Publication Reference	Study Design/Studied Population	Calcium and PTH Metabolism Amid the Diagnosis of Primary Hyperparathyroidism	Diagnostic Criteria of the Glucose Metabolism Anomalies
Barale 2024 [25]	Observational cross-sectional study N = 68 F:M = 48:20 (61% females) Mean age = 70 ± 9 y N1 = 17 with NCPHPT F:M = 12:5 (61% females) Mean age = 70 ± 9 y Mean BMI = 28 ± 4 kg/m^2^ N2 = 17 with HCPHPT F:M = 12:5 (61% females) Mean age = 70 ± 9 y Mean BMI = 29 ± 6 kg/m^2^ N3 = 34 controls F:M = 24:10 (61% females) Mean age = 70 ± 9 y Mean BMI = 29 ± 5 kg/m^2^ N1 were matched for age, sex, and BMI with N2 and N3	Mean ± SD N1 vs. N2 vs. N3: tCa (mmol/L): 2.4 ± 0.2 vs. 2.7 ± 0.2 vs. 2.3 ± 0.2 N1 vs. N2 *p* < 0.05 N1 vs. N3 *p* < 0.05 N2 vs. N3 *p* < 0.05 iCa (mmol/L): 1.2 ± 0.1 vs. 1.4 ± 0.1 vs. 1.1 ± 0.1 N1 vs. N2 *p* < 0.05 N1 vs. N3 *p* < 0.05 N2 vs. N3 *p* < 0.05 PO4 (mmol/L): 0.96 ± 0.15 vs. 0.87 ± 0.11 vs. 1.19 ± 0.27 N1 vs. N2 *p* < 0.05 N1 vs. N3 *p* < 0.05 N2 vs. N3 *p* < 0.05 25OHD (ng/mL): 38.8 ± 8.4 vs. 21.1 ± 9.9 vs. 23.3 ± 10.6 N1 vs. N2 *p* > 0.05 N1 vs. N3 *p* < 0.05 N2 vs. N3 *p* < 0.05 PTH (pg/mL): 60 ± 25 vs. 99 ± 42 vs. 29 ± 21 N1 vs. N2 *p* < 0.05 N1 vs. N3 *p* < 0.05 N2 vs. N3 *p* < 0.05 urinary Ca (mmol/24 h): 4.5 ± 2.7 vs. 8.5 ± 4 vs. 4.6 ± 4.5 N1 vs. N2 *p* < 0.05 N1 vs. N3 *p* > 0.05 N2 vs. N3 *p* < 0.05	Glucose disorders (IFG, IGT, T2DM) *
Govind 2024 [26]	Retrospective study N = 110 with PHPT F:M = 96:14 (87.3% females) Median (IQR) age = 57 (44–66.8) y Median (IQR) BMI = 27.4 (23.39–32.85) kg/m^2^ N1 = 84 with PHPT who underwent surgery, out of which: N2 = 14 with hungry bone syndrome Median (IQR) age = 39.5 (20.8–56) y Median (IQR) BMI = 22.7 (17.8–24.1) kg/m^2^ N3 = 70 without hungry bone syndrome Median (IQR) age = 27.4 (23.7–32.4) y Median (IQR) BMI = 27.4 (23.7–32.4) kg/m^2^	Mean ± SD or Median (IQR) N: tCa (mmol/L) = 2.87 ± 0.34 PO4 (mmol/L) = 0.87 ± 0.2 25OHD (nmol/L) = 42.9 (33.3–62.9) PTH (pmol/L) = 23.3 (16–45.4) urinary Ca mmol/24 h) = 3.3 (1.6–5.4)	Electronic health records
Misgar 2024 [27]	Cross-sectional study N = 103 with PHPT F:M = 84:19 (81.6% females) Mean age = 42.8 ± 14.73 y	Mean ± SD tCa (mg/dL) = 12.1 ± 1.34 PO4 (mg/dL) = 2.35 ± 0.613 25OHD (ng/mL) = 25.7 ± 15.12 PTH (pg/mL) = 332.9 ± 403.6 urinary Ca (mg/24 h) = 452.1 ± 256.4	NA
Nomine-Criqui 2024 [28]	Observational study N = 231 with PHPT who underwent surgery (out of which 41 with NCPHPT) F:M = 181:50 (78% females) Mean age = 63.2 ± 14.1 y Mean BMI = 26.8 ± 5.5 kg/m^2^	Mean ± SD tCa (mg/dL) = 10.99 ± 0.74 PO4 (mg/dL) = 2.59 ± 0.51 25OHD (ng/mL) = 23.8 ± 9.7 PTH (pmol/L) = 12.3 ± 9.5	DM as exclusion criterion Prediabetes (impaired fasting glucose): ADA criteria **: fasting glucose ≥1 g/L Insulin resistance: HOMA-IR *** > 2.5 EGIR ****: HOMA-IR > 1.8 for women and 2.12 for men
Zhang 2024 [29]	Population-based retrospective cohort study N = 16,494 F:M = 11,238:5256 (68.1% females) Mean age = 64.76 ± 16.22 y N1 = 2749 with PHPT F:M = 1873:876 (68.1% females) Mean age = 64.76 ± 16.22 y N2 = 13,745 index year, age and sex matched controls F:M = 9365:4380 (68.1% females) Mean age = 64.76 ± 16.22 y	Median (IQR) N1 vs. N2: tCa (mmol/L) = 2.63 (2.42–2.79) vs. 2.29 (2.2–2.37), *p* < 0.001 PO4 (mmol/L) = 0.84 (0.7–0.99) vs. 1.1 (0.97–1.23), *p* < 0.001	Incident T2DM: One of the following: HbA1c ≥ 6.5% or fasting plasma glucose ≥ 7 mmol/L or 2 h post-load with 75 g oral glucose (OGTT) ≥ 11.1 mmol/L or prescription of insulin or glucose-lowering drugs or diabetes codes from records
Bibik 2023 [30]	Prospective comparative study N1 = 24 with PHPT < 50 y (out of which 17 underwent PTx) F:M = 4:1 Median (IQR) age = 37 (33–41) y Median (IQR) BMI = 24.6 (22.5–26.5) kg/m^2^ N2 = 20 controls matched for sex, age and BMI Median (IQR) BMI = 23.9 (22.7–25.9) kg/m^2^	Median (IQR) N1 vs. N2 tCa albumin-adjusted (mmol/L): 2.73 (2.61–2.94) vs. 2.23 (2.15–2.28) *p* < 0.001 P (mmol/L): 0.76 (0.73–0.84) vs. 1.14 (1.09–1.25) *p* < 0.001 25OHD (ng/mL): 19.0 (13.3–21.9) vs. 20.8 (17.1–28.2) *p* = 0.154 PTH (pg/mL): 141 (111–228) vs. 39.9 (33.8–47.5) *p* < 0.001 N1 preoperative vs. post-operative—Median (IQR) tCa (mmol/L): 2.71 (2.61–2.91) vs. 2.18 (2.16–2.24) *p* < 0.001 P (mmol/L): 0.76 (0.72–0.83) vs. 0.94 (0.87–1.07) *p* = 0.001 25OHD (ng/mL): 20.5 (16.6–22.2) 30.4 (22.2–36.5) *p* = 0.004 PTH (pg/mL): 138 (106–210) vs. 38.8 (32.7–49.2) *p* < 0.001	DM as exclusion criterion
Dobreva 2023 [31]	Comparative study N = 838 with PHPT F:M = 775:63 (92.5% females) Median (IQR) age = 59 (51–66) y N1 = 150 with PHPT 18–49 y Median (IQR) BMI = 26 (22–29) kg/m^2^ N2 = 688 with PHPT ≥ 50 y Median (IQR) BMI = 28 (25–32) kg/m^2^ N1 vs. N2 BMI *p* < 0.001 Obesity N1 vs. N2: 24.2% vs. 35.9%, *p* = 0.006	Median (IQR) N1 vs. N2 tCa (mmol/L): 2.8 (2.7–3.0) vs. 2.8 (2.7–2.9) *p* = 0.034 iCa (mmol/L): 1.3 (1.2–1.4) vs. 1.3 (1.2–1.4) *p* = 0.236 Ca albumin-adjusted (mmol/L): 2.7 (2.6–2.9) vs. 2.7 (2.6–2.8) *p* = 0.079 P (mmol/L): 0.8 (0.7–0.9) vs. 0.9 (0.8–1.0) *p* < 0.001 25OHD (ng/mL): 21 (12–28) vs. 24 (17–34) *p* = 0.004 PTH (pg/mL): 132.3 (107–257) vs. 132 (99.9–209.5) *p* = 0.117 urinary Ca mmol/24 h): 9.3 (7.3–11.3) vs. 7.7 (5.2–10.5) *p* < 0.001	NA
Iglesias 2023 [32]	Observational, retrospective, non-interventional study N = 699,157 F:M = 382,597:316,560 (54.7% females) N1 = 6515 with PHPT F:M = 4261:2254 (65.4% females) Mean age = 67.6 ± 15.9 y N2 = 692,642 without PHPT F:M = 378,336:314,306 (54.62% females)	NA	Data from electronic health records extracted with artificial intelligence tools
Maldar 2023 [33]	Retrospective study N = 130 females with PHPT who underwent PTx Mean age = 52.1 ± 15.7 y N1 = 58 females premenopause Mean age = 37.9 ± 9.7 y N2 = 72 females post-menopause Mean age = 63.5 ± 8.8 y	Mean ± SD or Median (IQR) N vs. N1 vs. N2: tCa (mg/dL): 12.13 ± 1.27 vs. 12.35 ± 1.28 vs. 11.96 ± 1.22, *p* = 0.079 P (mg/dL): 2.5 ± 0.61 vs. 2.42 ± 0.54 vs. 2.56 ± 0.66, *p* = 0.195 25OHD (ng/mL): 22 (1.5–89) vs. 21 (1.5–89) vs. 22 (3.9–67.2), *p* = 0.322 PTH (pg/mL): 262 (42.7–3361) vs. 334 (44.6–2500) vs. 239 (42.7–3361), *p* = 0.051	Electronic health records
Şengül 2023 [34]	Retrospective observational study N = 128 with PHPT F:M = 106:22 (82.8% females) N1 = 66 with PHPT and 25OHD < 50 nmol/L F:M = 58:8 (82.8% females) Mean age = 55.5 ± 14.7 y Mean BMI = 28.4 ± 4.7 kg/m^2^ N2 = 62 with PHPT and 25OHD ≥ 50 nmol/L F:M = 48:14 (82.8% females) Mean age = 60.5 ± 13.1 y Mean BMI = 26.9 ± 3.5 kg/m^2^	Median (IQR) N1 vs. N2: tCa albumin-adjusted (mmol/L): 2.6 (2.5–3.8) vs. 2.6 (2.6–3.4), *p* = 0.4 PTH (ng/L): 138 (65–700) vs. 135 (72–1229), *p* = 0.8 urinary Ca (mg/24 h): 311 (100–922) vs. 282 (98–1300), *p* = 0.4	MetS: National Cholesterol Education Program Adult Treatment Panel III ***** Criteria T2DM: NA
Soto-Pedre 2023 [35]	Population-based study N1 = 11,616 with probable PHPT F:M = 7768:3848 (66.8% females) Mean age = 55.7 ± 25.5 y Mean BMI = 30 ± 6.2 kg/m^2^ N2 = 33,848 comparison cohort F:M = 23,304:11,544 (66.8% females) Mean age = 55.4 ± 23.1 y Mean BMI = 30.4 ± 6.2 kg/m^2^ N3 = 6795 with definite PHPT N1 vs. N2 BMI *p* < 0.01	Median (IQR) tCa albumin-adjusted (mmol/L): 2.7 (2.5–2.7) vs. 2.3 (2.2–2.4), *p* < 0.001 25OHD (nmol/L): 38 (24–63) vs. 40 (24–90), *p* = 0.281 PTH (pmol/L): 7.8 (4.1–13.2) vs. 6.4 (3.9–12.5), *p* < 0.01	Electronic health records based on ICD-10 codes
Al-Jehani 2022 [36]	Observational study N1 = 174 with PHPT F:M = 140:34 (80% females) Mean age = 61.8 ± 14.6 y Mean BMI = 27.3 ± 6.1 kg/m^2^ N2 = 171 controls F:M = 133:38 (% females) Mean age = 37.3 ± 8.2 y Mean BMI = 27.7 ± 5.9 kg/m^2^ Age in N1 vs. N2 *p* < 0.05 N3 = 35 with NCPHPT N4 = 93 with mild HCPHT N5 = 46 with classic HCPHPT	Mean ± SD N1: tCa (mg/dL) = 10.95 ± 0.93 P (mg/L) = 25.7 ± 4.8 25OHD (ng/mL) = 22.0 ± 16.9 PTH (pmol/L) = 14.9 ± 13.9	DM as exclusion criterion Insulin resistance: HOMA > 2.6 or fasting insulin > 12 mIU/L Impaired fasting glucose: ADA **: fasting glucose = 1.00–1.25 g/L Data were evaluated before PTx
Al-Saleh 2022 [37]	Multicentre observational study N = 205 with PHPT F:M = 163:42 (79.5% females) Mean age = 59.8 ± 15.5 y	Mean ± SD Ca albumin-adjusted (mmol/L): 2.8 ± 0.12 25OHD (nmol/L): 50.2 ± 1.8 PTH (pg/mL): 30 ± 20	Electronic health records
Frey 2022 [38]	Observational prospective study N = 139 with PHPT who underwent PTx F:M = 117:22 (84% females) Mean age = 63 ± 13.4 y Mean BMI = 25.9 ± 4.9 kg/m^2^ N1 = 19 with classic PHPT (Ca > 2.85 mmol/L) F:M = 13:6 (68% females) Mean age = 60 ± 15 y Mean BMI = 25.8 ± 4.1 kg/m^2^ N2 = 120 with mild PHPT (Ca ≤ 2.85 mmol/L) F:M = 104:16 (86.6% females) Mean age = 63.5 ± 13.1 y Mean BMI = 25.9 ± 5.1 kg/m^2^	Mean ± SD or Median (IQR) Before PTx vs. after PTx N: Ca albumin-adjusted (mmol/L): 2.68 ± 0.21 vs. 2.36 ± 0.13, *p* < 0.001 P (mmol/L): 0.78 ± 0.15 vs. 0.97 ± 0.17, *p* < 0.001 25OHD (ng/mL): 24.7 ± 10 vs. 30 ± 8.9, *p* < 0.001 PTH (pg/mL): 93.4 (77.6–121.1) vs. 47.4 (35.4–59.4), *p* < 0.001 urinary Ca (mmol/24 h): 4.95 ± 2.81 vs. 2.28 ± 1.36, *p* < 0.001 N1: tCa (mmol/L): 3.05 ± 0.25 vs. 2.39 ± 0.17, *p* < 0.001 P (mmol/L): 0.65 ± 0.20 vs. 0.87 ± 0.20, *p* < 0.001 25OHD (ng/mL): 22.3 ± 9.1 vs. 29.6 ± 10.9, *p* < 0.05 PTH (pg/mL): 145.4 (107.6–223.0) vs. 39.6 (27.8–70.4), *p* < 0.001 urinary Ca (mmol/24 h): 6.01 ± 3.59 vs. 2.10 ± 1.07, *p* < 0.001 N2: tCa (mmol/L): 2.62 ± 0.14 vs. 2.36 ± 0.12, *p* < 0.001 P (mmol/L): 0.80 ± 0.13 vs. 0.99 ± 0.16, *p* < 0.001 25OHD (ng/mL): 25.1 ± 10.1 vs. 30.1 ± 8.6, *p* < 0.001 PTH (pg/mL): 87.8 (76.0–112.5) vs. 47.5 (37.2–59.4), *p* < 0.001 urinary Ca (mmol/24 h): 4.77 ± 2.65 vs. 2.31 ± 1.41, *p* < 0.001	DM at baseline as exclusion criterion
Kumari 2022 [39]	Retrospective observational study N = 464 with PHPT F:M = 356:108 (76.7% females) Median age = 45 (34–55) y N1 = 54 with PHPT and T2DM F:M = 39:15 (72.22% females) Median (IQR) age = 54 (49–60) y Median (IQR) BMI = 26.5 (22.8–30.6) kg/m^2^ N2 = 409 with PHPT without T2DM F:M = 316:93 (77.26% females) Median (IQR) age = 43 (32–54) Median (IQR) BMI = 23.6 (20.3–26.8) kg/m^2^ N1 vs. N2 median age: *p* < 0.0001 median BMI: *p* = 0.003 N3 = 108 with PHPT without T2DM matched for age, sex and BMI with N1	Median (IQR) N1 vs. N3: tCa (mg/dL): 11.6 (10.98–12.61) vs. 11.7 (11.0–12.5), *p* = 0.93 PO4 (mg/dL): 2.5 (2.2–2.8) vs. 2.5 (2.2–3.1), *p* = 0.49 PTH (pg/mL): 203 (139.8–437.3) vs. 285 (166–692), *p* = 0.04 25OHD (ng/mL) 18.6 (12.52–33.46) vs. 22.0 (11.2–34.2), *p* = 0.82 urinary Ca (mg/24 h): 297 (128–460) vs. 218 (161–356), *p* = 0.48	T2DM: ADA criteria **
Nikooei 2021 [40]	Retrospective cohort study N = 65 with PHPT who underwent PTx F:M = 38:27 (58.5% females) Mean age = 45.44 ± 9.59 y Mean BMI = 26.65 ± 2.26 kg/m^2^	Mean ± SD Preoperative vs. post-operative tCa (mg/dL): 11.15 ± 0.39 vs. 9.91 ± 0.46, *p* = 0.0001 PTH (pg/mL): 112.49 ± 45.95 vs. 33.12 ± 9.83, *p* = 0.0001 Preoperative P (mg/dL): 2.85 ± 0.38	DM at baseline as exclusion criterion Insulin resistance ***: HOMA-IR ≥2.5 Moderate insulin resistance: HOMA-IR: 2.5–3.9 Severe insulin resistance: HOMA-IR > 3.9
Antonopoulou 2020 [41]	Observational pilot study N = 14 with PHPT F:M = 12:2 (85.71% females) Mean age = 52.93 ± 9.96 y Mean BMI = 27.19 ± 5.83 kg/m^2^	Mean ± SD Before PTx vs. after PTx tCa (mg/dL): 11.05 ± 0.49 vs. 9.11 ± 0.32, *p* < 0.001 Ca albumin-adjusted (mg/dL): 11.42 ± 0.53 vs. 9.4 ± 0.39, *p* < 0.001 P (mg/dL): 2.43 ± 0.64 vs. 3.06 ± 0.55, *p* = 0.007 25OHD (ng/mL): 24.47 ± 0.63 vs. 30.30 ± 11.97, *p* = 0.05 PTH (pmol/L): 11.62 ± 4.93 vs. 5.64 ± 1.53, *p* < 0.001	DM at baseline as exclusion criterion Insulin resistance ***: HOMA2-IR β-cell function: HOMA2-B% *** Insulin sensitivity: HOMA2-S% *** QUICKI ****** Matsuda ******* 75 g OGTT in all patients
Chen 2020 [42]	Cross-sectional study N = 392 with elevated PTH F:M = 220:172 (56.21% females) Mean age = 55.95 ± 1.28 y	Mean ± SD 25OHD (nmol/L): 50.91 ± 1.57 PTH (pg/mL): 87.35 ± 1.60	MetS score based on equations using waist circumference, high-density lipoprotein, triglycerides, fasting plasma glucose, systolic blood pressure ********
Karras SN 2020 [43]	Pilot study N = 62 N1 = 20 with NCPHPT and prediabetes F:M = 14:6 (70% females) Mean age = 66.2 ± 3.2 y Mean BMI = 28.6 ± 1.3 kg/m^2^ N2 = 42 controls with prediabetes F:M = 30:12 (71.43% females) Mean age = 63.9 ± 1 y Mean BMI = 30 ± 0.7 kg/m^2^	Mean ± SD N1 vs. N2 tCa (mg/dL): 9.8 ± 0.1 vs. 9.8 ± 0, *p* = 0.99 P (mg/dL): 3.4 ± 0.1 vs. 3.5 ± 0.0, *p* = 0.29 25OHD (ng/mL): 31.2 ± 1.3 vs. 26.3 ± 1.1, *p* = 0.09 PTH (pg/mL): 86.2 ± 3.2 vs. 29.2 ± 1.4, *p* < 0.01	DM at baseline as exclusion criterion Prediabetes: ADA criteria ** Insulin resistance: HOMA-IR Insulin secretion: HOMA-B% *** 75 g OGTT in all participants
Karras S 2020 [44]	Cohort study N1 = 16 with NCPHPT and prediabetes who underwent PTx F:M = 12:4 (75% females) Mean age = 58.9 ± 1 y Mean BMI = 28.1 ± 0.7 kg/m^2^ N2 = 16 with NCPHPT and prediabetes conservatively managed F:M = 11:5 (68.75% females) Mean age = 56.2 ± 3.2 y Mean BMI = 28.2 ± 1.3 kg/m^2^	Mean ± SD N1 baseline: tCa albumin-adjusted (mg/dL): 9.9 ± 0.0 P (mg/dL): 3.5 ± 0.0 25OHD (ng/mL): 36.3 ± 2.1 PTH (pg/mL): 94.2 ± 2.4 N2 baseline: tCa albumin-adjusted (mg/dL): 9.8 ± 0.1 P (mg/dL): 3.4 ± 0.1 25OHD (ng/mL): 33.2 ± 1.3 PTH (pg/mL): 96.2 ± 3.2 N1 after PTx: tCa albumin-adjusted (mg/dL): 9.1 ± 0.0 P (mg/dL): 3.9 ± 0.1 25OHD (ng/mL): 32.3 ± 3.1 PTH (pg/mL): 44.2 ± 1.4 N2 after 32 w: tCa albumin-adjusted (mg/dL): 9.7 ± 0.2 P (mg/dL): 3.6 ± 0.1 25OHD (ng/mL): 31.2 ± 1.9 PTH (pg/mL): 86.2 ± 2.2 N1 vs. N2 after 32 w: PTH: *p* = 0.02 Ca albumin-adjusted: *p* = 0.044 P: *p* = 0.031 25OHD: *p* = 0.383	DM at baseline as exclusion criterion Insulin resistance: HOMA-IR Insulin secretion: HOMA-B% *** 75-g OGTT in all participants Prediabetes: ADA criteria **: Impaired fasting glucose: fasting glucose: 101–125 mg/dL Impaired glucose tolerance: 2 h post-load glucose: 140–199 mg/dL HbA1c: 5.7–6.4%

Abbreviations: 25OHD = 25-hydroxyvitamin D; BMI = body mass index; DM = diabetes mellitus; F = female; HbA1c = glycated haemoglobin; HCPHPT = hypercalcemic primary hyperparathyroidism; HOMA-IR = Homeostatic Model Assessment for Insulin Resistance; HOMA-B% = Homeostasis Model Assessment of Beta-cell function; HOMA-S% = Homeostatic Model Assessment for Insulin Sensitivity; HR = hazard ratio; iCa = ionized serum calcium; ICD-10 = International Classification of Diseases Version 10; IFG = impaired fasting glycaemia; IGT = impaired glucose tolerance; IQR = interquartile range; IR = incidence rate; OGTT = oral glucose tolerance test; M = male; MetS = metabolic syndrome; N = number of patients; NA = not available; NCPHPT = normocalcemic primary hyperparathyroidism; PHPT = primary hyperparathyroidism; P = serum phosphorus; PO4 = serum phosphate; PTH = parathyroid hormone; PTx = parathyroidectomy; SD = standard deviation; T2DM = type 2 diabetes mellitus; tCa = total serum calcium; urinary Ca = calciuria; vs. = versus; y = years; (* WHO (World Health Organisation) criteria, please check [45]; ** ADA (American Diabetes Association) criteria, please check [46]; *** please check [47]; **** EGIR = European Group for the Study of Insulin Resistance, please check [48]; ***** please check [49]; ****** please check [50]; ******* please check [51]; ******** please check [52]).

**Table 2:** Analysis of T2DM prevalence and incidence in patients confirmed with primary hyperparathyroidism [25,26,27,29,31,32,33,34,35,36,37,38,39].

Reference	T2DM Prevalence/Incidence and Other Epidemiologic Findings
[25]	N1 vs. N2 vs. N3: T2DM: 12% vs. 35% vs. 12% N1 vs. N2 *p* < 0.05 N1 vs. N3 *p* > 0.05 N2 vs. N3 *p* < 0.05
[26]	In N: T2DM of 60.5% (66/110)
[27]	T2DM: 14.5% (15/103)
[29]	Incident diabetes N1: 433 vs. N2: 2110 IR of diabetes (95% CI) N1: 27.60 (25.00–30.30) per 1000 person-year N2: 23.90 (22.80–24.90) per 1000 person-year Risk of diabetes N1 vs. N2: Overall: HR (95% CI) = 1.15 (1.04–1.28), *p* = 0.007 18–35 y: HR (95% CI) = 2.59 (1.37–4.88, *p* = 0.004) 36–50 y: *p* = 0.123 51–65: *p* = 0.163 66–80: *p* = 0.408 >80 y: *p* = 0.338 Adjusted for screening frequency HR (95% CI) = 1.12 (1.01–1.24) N1 with Ca above median vs. below median (median Ca = 2.63 mmol/L): IR of diabetes: 28.80 vs. 26.50 per 1000 person-year, *p* = 0.011 HR (95% CI) = 1.44 (1.08–1.90)
[31]	N1 vs. N2: T2DM of 2.6% vs. 14.4%, *p* < 0.001
[32]	N1 vs. N2: T2DM of 31.3% vs. 9.3%, *p* < 0.001
[33]	N vs. N1 vs. N2: T2DM of 10.8% vs. 3.5% vs. 16.7%, *p* = 0.033
[34]	N1 vs. N2: T2DM of 19.7% vs. 8.1%, *p* = 0.05 25OHD (nmol/L): T2DM vs. without T2DM of 40 (13.5–136) vs. 47.8 (10–206.8), *p* = 0.82
[35]	N1 vs. N2: 8.1% (944/11,616) vs. 9.2% (3212/33,848), *p* < 0.001 HR (95% CI) of T2DM N1: 1.39 (1.26–1.54), *p* < 0.05 N3: 1.43 (1.28–1.60), *p* < 0.05 HR (95% CI) of T2DM in N1 (competing risk regression with death as competing event): 1.02 (0.95–1.1), *p* > 0.05 HR (95% CI) of T2DM in N1 (adjusted for vitamin D): 1.26 (1.07–1.48), *p* < 0.05
[36]	T2DM: 13.19% (31/235) + patients with T2DM were excluded from final analysis
[37]	T2DM: 4.9% (10/205)
[38]	T2DM: 4.01% (14/349) + patients with T2DM were excluded from final analysis
[39]	T2DM: 11.6% (54/464) N females vs. males: 11% vs. 13.9%, *p* = 0.51 Mean duration of T2DM before PHPT diagnosis: 6.8 ± 0.9 years Obesity N1 vs. N2: 48.1% vs. 30.2%, *p* = 0.008 N1 vs. N2: Bone disease: 37% vs. 47.2%, *p* = 0.29 Osteitis fibrosa cystica: 7.4% vs. 17.6%, *p* = 0.13 Nephrolithiasis: 18.5% vs. 36.1%, *p* = 0.03 Pancreatitis: 22.2% vs. 6.5%, *p* = 0.007 Psychiatric abnormalities: 20.4% vs. 9.3%, *p* = 0.08 Hypertension: 59.3% vs. 44.4%, *p* = 0.10 Anaemia: 51.9% vs. 50.9%, *p* = 0.96

Abbreviations: 25OHD = 25-hydroxyvitamin D; Ca = calcium; CI = confidence interval; HR = hazard ratio; IR = incidence rate; N = number of patients; PHPT = primary hyperparathyroidism; T2DM = type 2 diabetes mellitus; vs. = versus; y = years (please check Table 1 for sub-groups description amid studied population in each included study).

**Table 3:** Analysis of impaired glucose tolerance/impaired fasting glycaemia and insulin resistance in patients with primary hyperparathyroidism [28,31,36,40].

Reference	Prevalence of Prediabetes	Prevalence of Insulin Resistance
[28]	32% (75/231) Preoperative vs. 3 mo vs. 6 mo vs. 1 y: 32% vs. 39% vs. 35% vs. 35%, *p* = 0.555	HOMA-IR ≥ 2.5: 47% (108/231) EGIR: 67% (154/231) HOMA-IR ≥ 2.5 Preoperative vs. 3 mo vs. 6 mo vs. 1 y: 47% vs. 50% vs. 47% vs. 50%, *p* = 0.514 EGIR definition Preoperative vs. 3 mo vs. 6 mo vs. 1 y: 67% vs. 64% vs. 64% vs. 67%, *p* = 0.980
[31]	N1 vs. N2 Impaired glucose tolerance/impaired fasting glycaemia: 2% vs. 7.3%, *p* = 0.016	NA
[36]	N1 vs. N2: 36% (63/174) vs. 26% (44/171), *p* = 0.035 N3 vs. N4 vs. N5: 23% (8/35) vs. 39% (36/93) vs. 41% (19/46), *p* = 0.176	HOMA-IR > 2.6 N1 vs. N2: 45% (78/174) vs. 20% (34/171), *p* < 0.001 N3 vs. N4 vs. N5: 17% (6/35) vs. 43% (40/93) vs. 70% (32/46), *p* < 0.001
[40]	NA	Preoperative vs. postoperative 32.3% vs. 23.1%, *p* = 0.031

Abbreviations: EGIR = European Group for the Study of Insulin Resistance; HOMA-IR = Homeostatic Model Assessment for Insulin Resistance; mo = months; N = number of patients; NA = not available; vs. = versus; y = year (please check Table 1 for sub-groups description amid studied population in each included study).

**Table 4:** Glucose metabolism parameters in patients with primary hyperparathyroidism [25,26,28,30,31,36,38,39,40,41,43,44].

Reference	Fasting Glucose [Mean ± SD or Median (IQR)]	HbA1c (%) [Median (IQR)]	Fasting Insulin (Mean ± SD or Median (IQR)]
[25]	N1 vs. N3: 88 ± 11 vs. 95 ± 22 mg/dL, *p* > 0.05 N1 vs. N2: 88 ± 11 vs. 113 ± 31 mg/dL, *p* < 0.05 N2 vs. N3: 113 ± 31 vs. 95 ± 22 mg/dL, *p* < 0.05	NA	N1 vs. N3: 5.6 ± 2.9 vs. 11.7 ± 8.4 pmol/L, *p* > 0.05 N1 vs. N2: 5.6 ± 2.9 vs. 10.0 ± 6 pmol/L, *p* > 0.05 N2 vs. N3: 10.0 ± 6 vs. 11.7 ± 8.4 pmol/L, *p* > 0.05
[26]	NA	N2 vs. N3: 5.4 (5.3–5.8) vs. 6.3 (5.8–7.9), *p* = 0.008	NA
[28]	Preoperative vs. 3 mo vs. 6 mo vs. 1 y: 0.98 ± 0.11 vs. 0.98 ± 0.12 vs. 0.97 ± 0.12 vs. 0.98 ± 0.15 g/L, *p* = 0.573	NA	Preoperative vs. 3 mo vs. 6 mo vs. 1 y: 13.2 ± 9.9 vs. 13.2 ± 11 vs. 12.5 ± 8.7 vs. 12.3 ± 7.5 mUI/L, *p* = 0.0.82
[30]	N1 vs. N2: 5.04 (4.63–5.23) vs. 4.83 (4.50–5.20) mmol/L, *p* = 0.556 N1 preoperative vs. postoperative: 5.10 (4.81–5.24) vs. 4.69 (4.48–5.00) mmol/L, *p* = 0.031	N1 vs. N2: 5.30 (5.10–5.50) vs. 5.20 (5.10–5.50) *p* = 0.815 N1 preoperative vs. postoperative: 5.30 (5.10–5.50) vs. 5.60 (5.30–5.80) *p* = 0.001	N1 vs. N2 AUC Insulin phase 1: 61.9 (44.4–73.9) vs. 37.6 (36.1–42.6) *p* < 0.001 AUC Insulin phase 2: 160 (145–198) 132 (115–175) *p* = 0.019 N1 preoperative vs. postoperative AUC Insulin phase 1: 657 (426–862) vs. 501 (339–768), *p* = 0.163 AUC Insulin phase 2: 1121 (917–1320) vs. 982 (806–1375) *p* = 0.044
[31]	In patients without T2DM or IFG/IGT: 4.9 (4.6–5.3) vs. 5.2 (4.9–5.5) mmol/L, *p* < 0.001	In patients without T2DM or IFG/IGT: HbA1c (%) = 5.3 (5.0–5.4) vs. 5.6 (5.3–5.8) *p* = 0.002	NA
[36]	N1 vs. N2: 0.98 ± 0.13 vs. 0.93 ± 0.12 g/L, *p* < 0.001 N3 vs. N4 vs. N5: 0.961 ± 0.139 vs. 0.985 ± 0.125 vs. 0.995 ± 0.127 g/L, *p* = 0.473	NA	N1 vs. N2: 13.6 ± 12.3 vs. 8.07 ± 4.16 mUI/L, *p* < 0.001 N3 vs. N4 vs. N5: 8.92 ± 5 vs. 13.34 ± 13.76 vs. 17.73 ± 12 mUI/L, *p* = 0.005
[38]	Preoperative vs. 1 y postoperative N: 5.4 ± 0.6 vs. 5.2 ± 0.7 mmol/L, *p* < 0.001 N1: 5.5 ± 0.7 vs. 5.2 ± 0.9 mmol/L, *p* > 0.05 N2: 5.4 ± 0.6 vs. 5.2 ± 0.7 mmol/L, *p* < 0.01	NA	Preoperative vs. 1 y postoperative N: 9.4 ± 5.7 vs. 7.8 ± 4.9 mIU/L, *p* < 0.001 N1: 9.8 ± 5.2 vs. 7.8 ± 2.8 mIU/L, *p* > 0.05 N2: 9.3 ± 5.8 vs. 7.9 ± 5.1 mIU/L, *p* < 0.01
[39]	NA	N1: before PTx: 7.2% (6.4–8.9) vs. after PTx: 6.6% (6.2–7.8), *p* = 0.13	
[40]	Preoperative vs. postoperative 87.55 ± 7.94 vs. 85.83 ± 7.22 mg/dL, *p* = 0.01	Preoperative vs. postoperative: 5 (4.65–5.2) vs. 5 (4.45–5.1), *p* = 0.0001	Preoperative vs. postoperative: 10.4 (8.9–11.9) vs. 9.8 (8.2–11.07) pmol/L, *p* = 0.0001
[41]	Preoperative vs. postoperative 90.15 ± 16.28 vs. 92.08 ± 12.61 mg/dL, *p* = 0.42	NA	Preoperative vs. postoperative 8.70 ± 5.36 vs. 9.23 ± 4.57 μIU/mL, *p* = 0.42
[43]	N1 vs. N2: 105.6 ± 2.8 vs. 98.2 ± 1.8 mg/dL, *p* = 0.01	N1 vs. N2: 5.9 ± 0 vs. 5.9 ± 0, *p* = 0.44	N1 vs. N2: 14.0 ± 4.3 vs. 12.2 ± 1.1 μIU/mL, *p* = 0.53
[44]	N1 vs. N2: 119.4 ± 2.8 vs. 118.2 ± 1.8 mg/dL, *p* = 0.451 N1 preoperatively vs. postoperatively: 119.4 ± 2.8 vs. 111.2 ± 1.9 (−8.2 ± 0.6) mg/dL, *p* = 0.021 N2 baseline vs. 32 w: 118.2 ± 1.8 vs. 117.6 ± 2.3 (−0.6 ± 0.2) mg/dL, *p* = 0.031 N1 vs. N2 after 32 w: 111.2 ± 1.9 vs. 117.6 ± 2.3 (−6.4 ± 0.7) mg/dL, *p* = 0.02	N1 vs. N2: 5.84 ± 0.0 vs. 5.86 ± 0.0%, *p* = 0.415	N1 vs. N2: 11.0 ± 2.3 vs. 12.8 ± 1.4 µIU/mL, *p* = 0.731

Abbreviations: AUC = area under curve; IGT = impaired glucose tolerance; IFG = impaired fasting glycaemia; IQR = interquartile range; mo = month; N = number of patients; NA = not available; PTx = parathyroidectomy; SD = standard deviation; T2DM = type 2 diabetes mellitus; vs. = versus; y = year; w = week (please check Table 1 for sub-groups description amid studied population in each included study).

**Table 5:** 75-g oral glucose tolerance test in patients with primary hyperparathyroidism [41,43,44].

Reference	Main Findings During OGTT
[41]	Median ± SD or Mean (IQR) Preoperative vs. postoperative At 0 min Glucose: 90.15 ± 16.28 vs. 92.08 ± 12.61 mg/dL, *p* = 0.42 Insulin: 7.35 (11.27) vs. 8.30 (8.93) μIU/mL, *p* = 0.21 GLP-1: 74.73 ± 52.33 vs. 59.25 ± 25.67 pg/mL, *p* = 0.40 GIP: 8.7 ± 5.36 vs. 9.23 ± 4.57 pg/mL, *p* = 0.58 At 15 min Glucose: 139.5 ± 27.97 vs. 130.31 ± 24.25 mg/dL, *p* = 0.19 Insulin: 56.5 (53.45) vs. 45.85 (51.78) μIU/mL, *p* = 0.07 GLP-1: 67.06 (84.3) vs. 82.22 (72.88) pg/mL, *p* = 0.67 GIP: 3.52 (7.43) vs. 9.63 (17.86) pg/mL, *p* = 0.09 At 30 min Glucose: 147 ± 3.83 vs. 143 ± 37 mg/dL, *p* = 0.54 Insulin: 57 (77.9) vs. 66.35 (52.83) μIU/mL, *p* = 0.55 GLP-1: 55.08 (79.97) vs. 82.69 (73.46) pg/mL, *p* = 0.76 GIP: 9.99 (15.27) vs. 9.35 (17.99 pg/mL, *p* = 0.48 At 60 min Glucose: 152.50 ± 48.15 vs. 158.36 ± 47 mg/dL, *p* = 0.58 Insulin: 71.3 (93.4) vs. 70.50 (76.83) μIU/mL, *p* = 0.55 GLP-1: 63.06 ± 44.78 vs. 102.64 ± 40.19 pg/mL, *p* = 0.02 GIP: 9.25 (17.23) vs. 12.98 (14.45) pg/mL, *p* = 0.40 At 120 min Glucose: 113.38 ± 33.70 vs. 116.92 ± 35.96 mg/dL, *p* = 0.51 Insulin: 35.3 (63.25) vs. 38.25 (66.28) μIU/mL, *p* = 0.056 GLP-1: 71.20 ± 35.9 vs. 102.49 ± 40.02 pg/mL, *p* = 0.03 GIP: 20.18 (36.33) vs. 12.83 (15.18) pg/mL, *p* = 0.58
[43]	2-h post-load glucose: N1 vs. N2:157.2 ± 2.2 vs. 152.2 ± 2 mg/dL, *p* = 0.07
[44]	2-h post-load glucose: N1 vs. N2 baseline: 163.2 ± 3.2 vs. 167.2 ± 3.2 mg/dL, *p* = 0.371 N1 preoperatively vs. postoperatively: 163.2 ± 3.2 vs. 144.4 ± 3.2 (−18.8 ± 0.3) mg/dL, *p* = 0.041 N2 baseline vs. 32 w:167.2 ± 2.7 vs. 176.2 ± 3.2 (+9.0 ± 0.8) mg/dL, *p* = 0.781 N1 vs. N2 after 32 w:144.2 ± 3.2 vs. 176.2 ± 3.2 (−32 ± 0.4) mg/dL, *p* < 0.01

Abbreviations: GIP = gastric inhibitory polypeptide; GLP-1 = glucagon-like peptide 1; IQR = interquartile interval; h = hour; IQR = interquartile range; min = minute; N = number of patients; OGTT = oral glucose tolerance test; SD = standard deviation; vs. = versus; w = weeks; y = years (please check Table 1 for sub-groups description amid studied population in each included study).

**Table 6:** Insulin resistance and sensitivity in patients with PHPT [25,28,33,36,38,40,41,43].

Reference	HOMA-IR (Mean ± SD)	HOMA-B (%) (Mean ± SD)	HOMA-S (%) and Other Insulin Sensitivity Scores
[25]	N1 vs. N3: 1.1 ± 0.5 vs. 2.6 ± 2, *p* > 0.05 N1 vs. N2: 1.1 ± 0.5 vs. 2.7 ± 1.5 *p* > 0.05 N2 vs. N3: 2.7 ± 1.5 vs. 2.6 ± 2.0, *p* > 0.05	NA	NA
[28]	Preoperative vs. 3 mo vs. 6 mo vs. 1 y: 3.29 ± 2.79 vs. 3.28 ± 2.86 vs. 3.12 ± 2.49 vs. 3.07 ± 2.13, *p* = 0.514 N with prediabetes: Preoperative vs. 3 mo vs. 6 mo vs. 1 y: 4.79 ± 3.49 vs. 4.51 ± 3.54 vs. 4.20 ± 2.56 vs. 4.10 ± 2.38, *p* = 0.040 N without prediabetes: Preoperative vs. 3 mo vs. 6 mo vs. 1 y: 2.57 ± 2.03 vs. 2.66 ± 2.21 vs. 2.61 ± 2.29 vs. 2.58 ± 1.80, *p* = 0.933 N with insulin resistance: Preoperative vs. 3 mo vs. 6 mo vs. 1 y: 5.16 ± 3.14 vs. 4.83 ± 3.43 vs. 4.39 ± 2.77 vs. 4.31 ± 2.33, *p* = 0.002 N without insulin resistance: Preoperative vs. 3 mo vs. 6 mo vs. 1 y: 1.65 ± 0.48 vs. 1.91 ± 1.02 vs. 1.96 ± 1.45 vs. 1.99 ± 1.11, *p* = 0.001 N with insulin resistance according to EGIR: Preoperative vs. 3 mo vs. 6 mo vs. 1 y: 4.25 ± 2.97 vs. 4.06 ± 3.16 vs. 3.80 ± 2.60 vs. 3.76 ± 2.24, *p* = 0.016 N without insulin resistance according to EGIR: Preoperative vs. 3 mo vs. 6 mo vs. 1 y: 1.36 ± 0.34 vs. 1.76 ± 1.11 vs. 1.71 ± 1.44 vs. 1.69 ± 0.82, *p* = 0.001	Preoperative vs. 3 mo vs. 6 mo vs. 1 y: 140 ± 96 vs. 143 ± 117 vs. 135 ± 85 vs. 133 ± 74, *p* = 0.202	NA
[36]	N1 vs. N2: 3.39 ± 3.11 vs. 1.92 ± 1.16, *p* < 0.001 N3 vs. N4 vs. N5: 2.14 ± 1.29 vs. 3.28 ± 3.29 vs. 4.53 ± 3.51, *p* = 0.002	N1 vs. N2: 156 ± 262 vs. 100 ± 50, *p* = 0.006 N3 vs. N4 vs. N5: 105.9 ± 57.4 vs. 165.9 ± 348.7 vs. 176.4 ± 107.7, *p* = 0.447	NA
[38]	Preoperative vs. 1 y postoperative N: 2.3 ± 1.6 vs. 1.9 ± 1.3, *p* < 0.001 N1: 2.5 ± 1.6 vs. 1.9 ± 1.0 *p* > 0.05 N2: 2.3 ± 1.7 vs. 1.9 ± 1.3 *p* > 0.05	Preoperative vs. 1 y postoperative N: 99.4 ± 50.7 vs. 94.9 ± 55.9, *p* > 0.05 N1: 103.3 ± 54.1 vs. 102.2 ± 45.6 *p* > 0.05 N2: 98.9 ± 50.5 vs. 94.0 ± 57.2 *p* > 0.05	NA
[40]	Preoperative vs. postoperative 2.21 ± 0.69 vs. 2.02 ± 0.64, *p* = 0.0001	NA	NA
[41]	Preoperative vs. postoperative 1.14 ± 0.72 vs. 1.22 ± 0.61, *p* = 0.45	Preoperative vs. postoperative 97.53 ± 25.13 vs. 100.31 ± 28.22, *p* = 0.68	Preoperative vs. postoperative 127.74 ± 76.90 vs. 104.51 ± 48.15, *p* = 0.68 QUICKI: Preoperative vs. postoperative 0.36 ± 0.04 vs. 0.34 ± 0.03, *p* = 0.08 Matsuda Index: Preoperative vs. postoperative 6.34 ± 3.7 vs. 5.27 ± 2.44, *p* = 0.06
[43]	N1 vs. N2 3.7 ± 1.2 vs. 2.9 ± 0.2, *p* = 0.48	N1 vs. N2 117.8 ± 31.8 vs. 146.9 ± 22.0, *p* = 0.14	NA
[44]	N1 vs. N2 3.1 ± 1.2 vs. 2.9 ± 0.2, *p* = 0.211	N1 vs. N2 112.9 ± 31.8 vs. 116.9 ± 21.0, *p* = 0.314	NA

Abbreviations: EGIR = European Group for the Study of Insulin Resistance; HOMA-IR = Homeostatic Model Assessment for Insulin Resistance; HOMA-B% = Homeostasis Model Assessment of Beta-cell function; HOMA-S% = Homeostatic Model Assessment for Insulin Sensitivity; IQR = interquartile range; mo = month; N = number of patients; NA = not available; QUICKI = Quantitative Insulin Sensitivity Check Index; SD = standard deviation; (please check Table 1 for sub-groups description amid studied population in each included study).

**Table 7:** Metabolic syndrome in patients with primary hyperparathyroidism [34,42].

Reference	Main Findings
[34]	N1 vs. N2: MetS: 40.9% vs. 24.2%, *p* = 0.04 Median (IQR) 25OHD levels: MetS vs. without MetS: 40.8 (10–150.8) vs. 52.8 (10–206.8), *p* = 0.52
[42]	Mean MetS severity score: 94.78 ± 4.53 Statistically significant correlation between PTH and MetS severity scores in: Overall: β = 0.399, *p* = 0.030 With moderate physical activity: β = 0.413, *p* = 0.045 Without vitamin D supplementation: β = 0.524, *p* = 0.028 With high protein intake: β = 0.586, *p* = 0.03 With vitamin D deficiency: β = 0.456, *p* = 0.014

Abbreviations: 25OHD = 25-hydroxyvitamin D; BMI = body mass index; F = female; M = male; MetS = metabolic syndrome; N = number; NA = not available; PHPT = primary hyperparathyroidism; PTx = parathyroidectomy; SD = standard deviation; vs. = versus; y = years (please check Table 1 for sub-groups description amid studied population in each included study).

## Data Availability

Not applicable.

## Abbreviations

The following abbreviations are used in this manuscript:

ADA	American Diabetes Association
AUC	aria under curve
BMI	body mass index
CI	confidence interval
EGIR	European Group for the Study of Insulin Resistance
F	female
F:M	female-to-male ratio
GLP-1	glucagon-like peptide 1
GIP	gastric inhibitory polypeptide
iCa	ionized serum calcium
IQR	interquartile range
IGT	impaired glucose tolerance
IR	incidence rate
IDF	International Diabetes Federation
IFG	impaired fasting glycaemia
IGT	impaired glucose tolerance
M	male
HCPHPT	hypercalcaemic primary hyperparathyroidism
HOMA-IR	Homeostatic Model Assessment for Insulin Resistance
HOMA-B%	Homeostasis Model Assessment of Beta-cell function
HOMA-S%	Homeostatic Model Assessment for Insulin Sensitivity
HbA1c	glycated haemoglobin A1c
HR	hazard ratio
25OHD	25-hydroxyvitamin D
MetS	metabolic syndrome
NCPHPT	normocalcemic primary hyperparathyroidism
N	number of patients
n	number of studies
NA	not available
OGTT	75-g oral glucose tolerance test
QUICKI	Quantitative Insulin Sensitivity Check Index
PHPT	primary hyperparathyroidism
PTH	parathyroid hormone
PTx	parathyroidectomy
P	serum phosphorus
PO4	serum phosphate
SD	standard deviation
T2DM	type 2 diabetes mellitus
tCa	total serum calcium
vs.	versus
y	years
WHO	World Health Organisation

## Appendix A

**Table A1:** Parathyroidectomy and glucose metabolism findings [26,28,30,38,40,41,44].

Reference	Main Findings
[26]	Median (IQR) for HbA1c (%) in N2 vs. N3: 5.4 (5.3–5.8) vs. 6.3 (5.8–7.9); *p* = 0.008
[28]	Preoperative vs. 3 mo vs. 6 mo vs. 1 y: Prevalence of prediabetes:32% vs. 39% vs. 35% vs. 35%, *p* = 0.555 Fasting glucose: 0.98 ± 0.11 vs. 0.98 ± 0.12 vs. 0.97 ± 0.12 vs. 0.98 ± 0.15 g/L, *p* = 0.573 Fasting insulin: 13.2 ± 9.9 vs. 13.2 ± 11 vs. 12.5 ± 8.7 vs. 12.3 ± 7.5 mUI/L, *p* = 0.0.82 HOMA-IR: Overall: 3.29 ± 2.79 vs. 3.28 ± 2.86 vs. 3.12 ± 2.49 vs. 3.07 ± 2.13, *p* = 0.514 N with prediabetes: 4.79 ± 3.49 vs. 4.51 ± 3.54 vs. 4.20 ± 2.56 vs. 4.10 ± 2.38, *p* = 0.040 N without prediabetes: 2.57 ± 2.03 vs. 2.66 ± 2.21 vs. 2.61 ± 2.29 vs. 2.58 ± 1.80, *p* = 0.933 N with IR: 5.16 ± 3.14 vs. 4.83 ± 3.43 vs. 4.39 ± 2.77 vs. 4.31 ± 2.33, *p* = 0.002 N without IR: 1.65 ± 0.48 vs. 1.91 ± 1.02 vs. 1.96 ± 1.45 vs. 1.99 ± 1.11, *p* = 0.001 N with IR according to EGIR: 4.25 ± 2.97 vs. 4.06 ± 3.16 vs. 3.80 ± 2.60 vs. 3.76 ± 2.24, *p* = 0.016 N without IR according to EGIR: 1.36 ± 0.34 vs. 1.76 ± 1.11 vs. 1.71 ± 1.44 vs. 1.69 ± 0.82, *p* = 0.001 HOMA-B% Preoperative vs. 3 mo vs. 6 mo vs. 1 y: 140 ± 96 vs. 143 ± 117 vs. 135 ± 85 vs. 133 ± 74, *p* = 0.202 Correlations between change in HOMA-IR (preoperative vs. post-operative) and Ca: r = −0.173, *p* = 0.008 No correlation with the following: PTH: r = −0.006, *p* = 0.927 25OHD: r = −0.034, *p* = 0.607
[30]	N1 preoperative vs. post-operative Fasting plasma glucose: 5.10 (4.81–5.24) vs. 4.69 (4.48–5.00) mmol/L, *p* = 0.031 HbA1c (%): 5.30 (5.10–5.50) vs. 5.60 (5.30–5.80), *p* = 0.001 AUC Insulin phase 1:657 (426–862) vs. 501 (339–768), *p* = 0.163 AUC Insulin phase 2: 1121 (917–1320) vs. 982 (806–1375) *p* = 0.044 Peptide C: AUC phase 1: 63.2 (47.5–73.5) vs. 53.9 (44.2–71.0) *p* = 0.679 AUC phase 2: 161 (149–193) vs. 169 (139–196), *p* = 0.737
[38]	Preoperative vs. 1 y post-operative Fasting glucose: N: 5.4 ± 0.6 vs. 5.2 ± 0.7 mmol/L, *p* < 0.001 N1: 5.5 ± 0.7 vs. 5.2 ± 0.9 mmol/L, *p* > 0.05 N2: 5.4 ± 0.6 vs. 5.2 ± 0.7 mmol/L, *p* < 0.01 Fasting insulin: N: 9.4 ± 5.7 vs. 7.8 ± 4.9 mIU/L, *p* < 0.001 N1: 9.8 ± 5.2 vs. 7.8 ± 2.8 mIU/L, *p* > 0.05 N2: 9.3 ± 5.8 vs. 7.9 ± 5.1 mIU/L, *p* < 0.01 HOMA-IR N: 2.3 ± 1.6 vs. 1.9 ± 1.3, *p* < 0.001 N1: 2.5 ± 1.6 vs. 1.9 ± 1.0 *p* > 0.05 N2: 2.3 ± 1.7 vs. 1.9 ± 1.3 *p* > 0.05 HOMA-B% N: 99.4 ± 50.7 vs. 94.9 ± 55.9, *p* > 0.05 N1: 103.3 ± 54.1 vs. 102.2 ± 45.6 *p* > 0.05 N2: 98.9 ± 50.5 vs. 94.0 ± 57.2 *p* > 0.05 Adiponectin N: 6.2 ± 3.6 vs. 7.2 ± 3.9 μg/mL, *p* < 0.001 N1: 5.8 ± 3.5 vs. 6.7 ± 3.3 μg/mL, *p* < 0.01 N2: 6.3 ± 3.6 vs. 7.3 ± 4.0 μg/mL, *p* < 0.001 No correlation between Change in Ca albumin-adjusted and change in: Fasting plasma glucose (N: *p* = 0.96, N1: *p* = 0.86, N2: *p* = 0.62) Fasting plasma insulin (N: *p* = 0.90, N1: *p* = 0.38, N2: *p* = 0.69) HOMA-IR (N: *p* = 0.80, N1: *p* = 0.58, N2: *p* = 0.55) HOMA-B (N: *p* = 0.56, N1: *p* = 0.34, N2: *p* = 0.7) Adiponectin (N: *p* = 0.32, N1: *p* = 0.35, N2: *p* = 0.76) Change in PTH and change in: Fasting plasma glucose (N: *p* = 0.78, N1: *p* = 0.49, N2: *p* = 0.88) Fasting plasma insulin (N: *p* = 0.65, N1: *p* = 0.76, N2: *p* = 0.78) HOMA-IR (N: *p* = 0.76, N1: *p* = 0.88, N2: *p* = 0.88) HOMA-B (N: *p* = 0.84, N1: *p* = 0.094, N2: *p* = 0.78) Adiponectin (N: *p* = 0.70, N1: *p* = 0.88, N2: *p* = 0.97)
[40]	Preoperative vs. post-operative Prevalence of insulin resistance: 32.3% vs. 23.1%, *p* = 0.031 Fasting plasma glucose: 87.55 ± 7.94 vs. 85.83 ± 7.22 mg/dL, *p* = 0.01 HbA1c (%) Median (25–75 percentile): 5 (4.65–5.2) vs. 5 (4.45–5.1), *p* = 0.0001 Insulin: Median (25–75 percentile) insulin: 10.4 (8.9–11.9) vs. 9.8 (8.2–11.07) pmol/L, *p* = 0.0001 HOMA-IR: 2.21 ± 0.69 vs. 2.02 ± 0.64, *p* = 0.0001
[41]	Preoperative vs. post-operative: Fasting plasma glucose: 90.15 ± 16.28 vs. 92.08 ± 12.61 mg/dL, *p* = 0.42 Fasting insulin: 8.70 ± 5.36 vs. 9.23 ± 4.57 μIU/mL, *p* = 0.42 HOMA-IR: 1.14 ± 0.72 vs. 1.22 ± 0.61, *p* = 0.45 HOMA-B%: 97.53 ± 25.13 vs. 100.31 ± 28.22, *p* = 0.68 HOMA-S%: 127.74 ± 76.90 vs. 104.51 ± 48.15, *p* = 0.68 QUICKI: 0.36 ± 0.04 vs. 0.34 ± 0.03, *p* = 0.08 Matsuda Index: 6.34 ± 3.7 vs. 5.27 ± 2.44, *p* = 0.06 GLP-1: 74.73 ± 52.33 vs. 59.25 ± 25.67 pg/mL, *p* = 0.58 GIP: 3.45 (7.43) vs. 9.84 (29.59) pg/mL, *p* = 0.26 Correlations before PTx Calcium and HOMA-S% r = −0.59, *p* = 0.03 PTH and: GLP-1 r = 0.79, *p* = 0.02 HOMA-B% r = 0.74, *p* = 0.002 HOMA-IR r = 0.43, *p* = 0.13 Matsuda index r = −0.3, *p* = 0.30 QUICKI r = −0.32, *p* = 0.26 Correlations after PTx Calcium and HOMA-S% r = 0.06, *p* = 0.84 PTH and: GLP-1 r = −0.5, *p* = 0.71 HOMA-B% r = 0.55, *p* = 0.04 HOMA-IR r = 0.56, *p* = 0.04 Matsuda index r = −0.58, *p* = 0.03 QUICKI r = −0.41, *p* = 0.04 Similar glucose and insulin response to OGTT before and after PTx
[44]	N1 vs. N2: Fasting plasma glucose: 119.4 ± 2.8 vs. 118.2 ± 1.8 mg/dL, *p* = 0.451 2 h post-load: 163.2 ± 3.2 vs. 167.2 ± 3.2 mg/dL, *p* = 0.371 HbA1c: 5.84 ± 0.0 vs. 5.86 ± 0.0%, *p* = 0.415 Fasting insulin: 11.0 ± 2.3 vs. 12.8 ± 1.4 µIU/mL, *p* = 0.731 N1 preoperatively vs. post-operatively: Fasting plasma glucose: 119.4 ± 2.8 vs. 111.2 ± 1.9 (−8.2 ± 0.6) mg/dL, *p* = 0.021 2 h post-load: 163.2 ± 3.2 vs. 144.4 ± 3.2 (−18.8 ± 0.3) mg/dL, *p* = 0.041 N1 preoperatively vs. N2 32 w: Fasting plasma glucose: 111.2 ± 1.9 vs. 117.6 ± 2.3 (−6.4 ± 0.7) mg/dL, *p* = 0.02 2 h post-load: 144.2 ± 3.2 vs 176.2 ± 3.2 (−32 ± 0.4) mg/dL, *p* < 0.01

Abbreviations: AUC = area under curve; BMI = body mass index; F = female; HbA1c = glycated hemoglobin; HCPHPT = hypercalcemic primary hyperparathyroidism; HOMA-B% = Homeostasis Model Assessment of Beta-cell function; HOMA-IR = Homeostatic Model Assessment for Insulin Resistance; IQR = interquartile range; M = male; mo = months; M-value = glucose uptake by tissues; N = number; NA = not available; NCPHPT = normocalcemic primary hyperparathyroidism; OGTT = oral glucose tolerance test; QUICKI = Quantitative Insulin Sensitivity Check Index; PHPT = primary hyperparathyroidism; PTx = parathyroidectomy; T2DM = type 2 diabetes mellitus; vs. = versus; w = weeks; y = years (please check Table 1 for sub-group descriptions amid studied population in each included study).

**Table A2:** Adiponectin, leptin, GIP, and GLP-1 in patients with primary hyperparathyroidism [30,38,41].

Reference	Adiponectin	Leptin	Fasting GLP-1	Fasting GIP
[30]	Median (IQR) N1 vs. N2 7.22 (4.56–8.79) vs. 7.23 (4.81–10.8) μg/mL, *p* = 0.849 N1 preoperative vs. postoperative 8.08 (6.27–9.71) vs. 7.10 (3.98–10.3) μg/mL, *p* = 0.059	Median (IQR) N1 vs. N2 12.5 (4.74–18.8) vs. 7.09 (6.28–11.7) μg/L, *p* = 0.247 N1 preoperative vs. postoperative 10.8 (4.36–17.6) vs. 12.1 (4.09–24.8) μg/L, *p* = 0.123	NA	NA
[38]	Mean ± SD Preoperative vs. 1 y postoperative N: 6.2 ± 3.6 vs. 7.2 ± 3.9 μ, *p* < 0.001 N1: 5.8 ± 3.5 vs. 6.7 ± 3.3 μg/mL, *p* < 0.01 N2: 6.3 ± 3.6 vs. 7.3 ± 4.0 μg/mL, *p* < 0.001	NA	NA	NA
[41]	NA	NA	Mean ± SD Preoperative vs. postoperative 74.73 ± 52.33 vs. 59.25 ± 25.67 pg/mL, *p* = 0.58	Median (IQR) Preoperative vs. postoperative 3.45 (7.43) vs. 9.84 (29.59) pg/mL, *p* = 0.26

Abbreviations: BMI = body mass index; F = female; GIP = gastric inhibitory polypeptide; GLP-1 = glucagon-like peptide 1; IQR = interquartile range; M = male; N = number; NA = not available; PHPT = primary hyperparathyroidism; PTx = parathyroidectomy; SD = standard deviation; vs. = versus; y = years (please check Table 1 for sub-group descriptions amid studied population in each included study).

**Table A3:** Glucose metabolism in patients with normocalcemic primary hyperparathyroidism [25,36,43,44].

Reference	Main Findings
[25]	Prevalence of T2DM: N1 vs. N2 vs. N3: 12% vs. 35% vs. 12% N1 vs. N2 *p* < 0.05 N1 vs. N3 *p* > 0.05 N2 vs. N3 *p* < 0.05 Glucose disorders: N1 vs. N3: 6% vs. 9%, *p* > 0.05 N1 vs. N2: 6% vs. 41%, *p* < 0.05 Glucose (Mean ± SD): N1 vs. N3: 88 ± 11 vs. 95 ± 22 mg/dL, *p* > 0.05 N1 vs. N2: 88 ± 11 vs. 113 ± 31 mg/dL, *p* < 0.05 N2 vs. N3: 113 ± 31 vs. 95 ± 22 mg/dL, *p* < 0.05 Insulin (Mean ± SD): N1 vs. N3: 5.6 ± 2.9 vs. 11.7 ± 8.4 pmol/L, *p* > 0.05 N1 vs. N2: 5.6 ± 2.9 vs. 10.0 ± 6 pmol/L, *p* > 0.05 N2 vs. N3: 10.0 ± 6 vs. 11.7 ± 8.4 pmol/L, *p* > 0.05 HOMA-IR (Mean ± SD): N1 vs. N3: 1.1 ± 0.5 vs. 2.6 ± 2, *p* > 0.05 N1 vs. N2: 1.1 ± 0.5 vs. 2.7 ± 1.5 *p* > 0.05 N2 vs. N3: 2.7 ± 1.5 vs. 2.6 ± 2.0, *p* > 0.05 Correlation between tCa and glucose in N1 + N2: r = 0.46, *p* < 0.05 No correlation between tCa or PTH and glucose in N2 No correlation between PTH or iCa and glucose in N1 + N2
[36]	Prevalence of prediabetes: N3 vs. N4 vs. N5: 23% (8/35) vs. 39% (36/93) vs. 41% (19/46), *p* = 0.176 Prevalence of insulin resistance (HOMA-IR > 2.6): N3 vs. N4 vs. N5: 17% (6/35) vs. 43% (40/93) vs. 70% (32/46), *p* < 0.001 Fasting glucose: N3 vs. N4 vs. N5: 0.961 ± 0.139 vs. 0.985 ± 0.125 vs. 0.995 ± 0.127 g/L, *p* = 0.473 Fasting insulin: N3 vs. N4 vs. N5: 8.92 ± 5 vs. 13.34 ± 13.76 vs. 17.73 ± 12 mUI/L, *p* = 0.005 HOMA-IR N3 vs. N4 vs. N5: 2.14 ± 1.29 vs. 3.28 ± 3.2 vs. 4.53 ± 3.51, *p* = 0.002 HOMA-B% N3 vs. N4 vs. N5: 105.9 ± 57.4 vs. 165.9 ± 348.7 vs. 176.4 ± 107.6, *p* = 0.447
[43]	N1 vs. N2 Fasting plasma glucose: 105.6 ± 2.8 vs. 98.2 ± 1.8 mg/dL, *p* = 0.01 2 h post-load: 157.2 ± 2.2 vs. 152.2 ± 2 mg/dL, *p* = 0.07 HbA1c: 5.9 ± 0 vs. 5.9 ± 0, *p* = 0.44 Fasting insulin: 14.0 ± 4.3 vs. 12.2 ± 1.1 μIU/mL, *p* = 0.53 HOMA-IR: 3.7 ± 1.2 vs. 2.9 ± 0.2, *p* = 0.48 HOMA-B%: 117.8 ± 31.8 vs. 146.9 ± 22.0, *p* = 0.14
[44]	N1 preoperatively vs. post-operatively: Fasting plasma glucose: 119.4 ± 2.8 vs. 111.2 ± 1.9 (−8.2 ± 0.6) mg/dL, *p* = 0.021 2 h post-load: 163.2 ± 3.2 vs. 144.4 ± 3.2 (−18.8 ± 0.3) mg/dL, *p* = 0.041

Abbreviations: BMI = body mass index; F = female; HbA1c = glycated hemoglobin; HCPHPT = hypercalcemic primary hyperparathyroidism; HOMA-B% = Homeostasis Model Assessment of Beta-cell function; HOMA-IR = Homeostatic Model Assessment for Insulin Resistance; M = male; N = number; NA = not available; NCPHPT = normocalcemic primary hyperparathyroidism; OGTT = oral glucose tolerance test; PHPT = primary hyperparathyroidism; PTx = parathyroidectomy; SD = standard deviation; T2DM = type 2 diabetes mellitus; vs. = versus; y = years (please check Table 1 for sub-groups description amid studied population in each included study).

## References

[1] Silva B.C., Cusano N.E., Bilezikian J.P. (2024). Primary hyperparathyroidism. Best. Pract. Res. Clin. Endocrinol. Metab..

[2] Kurtom S., Carty S.E. (2024). Primary Hyperparathyroidism: Part One: Evaluation. Surg. Clin. N. Am..

[3] Nabata K.J., Wiseman J.J., Wiseman S.M. (2023). Normohormonal primary hyperparathyroidism: A systematic review and meta-analysis. Am. J. Surg..

[4] El-Hajj Fuleihan G., Chakhtoura M., Cipriani C., Eastell R., Karonova T., Liu J.M., Minisola S., Mithal A., Moreira C.A., Peacock M. (2022). Classical and Nonclassical Manifestations of Primary Hyperparathyroidism. J. Bone Miner. Res..

[5] Iwanowska M., Kochman M., Szatko A., Zgliczyński W., Glinicki P. (2024). Bone Disease in Primary Hyperparathyroidism-Changes Occurring in Bone Metabolism and New Potential Treatment Strategies. Int. J. Mol. Sci..

[6] Kochman M. (2023). Primary hyperparathyroidism: Clinical manifestations, diagnosis and evaluation according to the Fifth International Workshop guidelines. Reumatologia.

[7] Abel E.D., Gloyn A.L., Evans-Molina C., Joseph J.J., Misra S., Pajvani U.B., Simcox J., Susztak K., Drucker D.J. (2024). Diabetes mellitus-Progress and opportunities in the evolving epidemic. Cell.

[8] Wong N.D., Sattar N. (2023). Cardiovascular risk in diabetes mellitus: Epidemiology, assessment and prevention. Nat. Rev. Cardiol..

[9] Zhou B., Rayner A.W., Gregg E.W., Sheffer K.E., Carrillo-Larco R.M., Bennett J.E., Shaw J.E., Paciorek C.J., Singleton R.K., Pires A.B. (2024). Worldwide trends in diabetes prevalence and treatment from 1990 to 2022: A pooled analysis of 1108 population-representative studies with 141 million participants. Lancet.

[10] Ong K.L., Stafford L.K., McLaughlin S.A., Boyko E.J., Vollset S.E., Smith A.E., Dalton B.E., Duprey J., Cruz J.A., Hagins H. (2023). Global, regional, and national burden of diabetes from 1990 to 2021, with projections of prevalence to 2050: A systematic analysis for the Global Burden of Disease Study 2021. Lancet.

[11] Barnett M.J. (2023). Association Between Primary Hyperparathyroidism and Secondary Diabetes Mellitus: Findings from a Scoping Review. Cureus.

[12] Carsote M., Paduraru D.N., Nica A.E., Valea A. (2016). Parathyroidectomy: Is vitamin D a player for a good outcome?. J. Med. Life.

[13] Kumar A., Singh S. (2015). Parathyroidectomy Ameliorates Glucose and Blood Pressure Control in a Patient with Primary Hyperparathyroidism, Type 2 Diabetes, and Hypertension. Clin. Med. Insights Endocrinol. Diabetes.

[14] Krumeich L.N., Santos A., Fraker D.L., Kelz R.R., Wachtel H. (2024). Modern Trends for Primary Hyperparathyroidism: Intervening on Less Biochemically Severe Disease. J. Surg. Res..

[15] Kurtom S., Carty S.E. (2024). Primary Hyperparathyroidism: Part Two: Surgical Management. Surg. Clin. N. Am..

[16] Dumitru N., Carsote M., Cocolos A., Petrova E., Olaru M., Dumitrache C., Ghemigian A. (2019). The Link Between Bone Osteocalcin and Energy Metabolism in a Group of Postmenopausal Women. Curr. Health Sci. J..

[17] Bilezikian J.P., Khan A.A., Silverberg S.J., Fuleihan G.E., Marcocci C., Minisola S., Perrier N., Sitges-Serra A., Thakker R.V., Guyatt G. (2022). Evaluation and Management of Primary Hyperparathyroidism: Summary Statement and Guidelines from the Fifth International Workshop. J. Bone Miner. Res..

[18] Vasiliu O., Panea C.A., Mangalagiu A.G., Petrescu B.M., Cândea C.A., Manea M.M., Ciobanu A.M., Sîrbu C.A., Mitrică M. (2025). Case Management of Delirium in Patients with Major Neurocognitive Disorders. Rom. J. Mil. Med..

[19] Wilhelm S.M., Wang T.S., Ruan D.T., Lee J.A., Asa S.L., Duh Q.Y., Doherty G.M., Herrera M.F., Pasieka J.L., Perrier N.D. (2016). The American Association of Endocrine Surgeons Guidelines for Definitive Management of Primary Hyperparathyroidism. JAMA Surg..

[20] Ciuche A., Nistor C., Pantile D., Marin D., Tudose A. (2011). *Spontaneous pneumothorax* in a case of pulmonary langerhans cell histiocytosis. Maedica.

[21] Yavari M., Feizi A., Haghighatdoost F., Ghaffari A., Rezvanian H. (2021). The influence of parathyroidectomy on cardiometabolic risk factors in patients with primary hyperparathyroidism: A systematic review and meta-analysis. Endocrine.

[22] Beysel S., Caliskan M., Kizilgul M., Apaydin M., Kan S., Ozbek M., Cakal E. (2019). Parathyroidectomy improves cardiovascular risk factors in normocalcemic and hypercalcemic primary hyperparathyroidism. BMC Cardiovasc. Disord..

[23] Godang K., Lundstam K., Mollerup C., Fougner F., Pernow Y., Nordenström J., Rosén T., Jansson S., Hellström M., Bollerslev J. (2018). The effect of surgery on fat mass, lipid and glucose metabolism in mild primary hyperparathyroidism. Endocr. Connect..

[24] Wu C., Gillis A., Lindeman B., Chen H., Fazendin J. (2024). Normocalcemic primary hyperparathyroidism: From pathophysiology to clinical management. Am. J. Surg..

[25] Barale M., Maiorino F., Pusterla A., Fraire F., Sauro L., Presti M., Sagone N., Ghigo E., Arvat E., Procopio M. (2024). Normocalcemic primary hyperparathyroidism is not associated with cardiometabolic alterations. Endocrine.

[26] Govind K., Paruk I.M., Motala A.A. (2024). Characteristics, management and outcomes of primary hyperparathyroidism from 2009 to 2021: A single centre report from South Africa. BMC Endocr. Disord..

[27] Misgar R.A., Wani M., Qadir A., Chhabra A. (2024). Clinical, Biochemical and Surgical Outcomes of Primary Hyperparathyroidism in the Present Era: A Prospective Study From a Tertiary Care Hospital. Cureus.

[28] Nomine-Criqui C., Bihain F., Nguyen-Thi P.L., Scheyer N., Demarquet L., Klein M., Guerci B., Brunaud L. (2024). Patients with prediabetes improve insulin resistance after surgery for primary hyperparathyroidism. Surgery.

[29] Zhang Y., Wu H., Yang A., YHNg N., Zhang X., Lau E.S.H., Chow E.W.K., Kong A.P.S., Chow E.Y.K., Chan J.C.N. (2024). Higher risk of incident diabetes among patients with primary hyperparathyroidism. Clin. Endocrinol..

[30] Bibik E.E., Dobreva E.A., Elfimova A.R., Miliutina A.P., Eremkina A.K., Gorbacheva A.M., Krupinova J.A., Koksharova E.O., Sklyanik I.A., Mayorov A.Y. (2023). Primary hyperparathyroidism in young patients is associated with metabolic disorders: A prospective comparative study. BMC Endocr. Disord..

[31] Dobreva E.A., Gorbacheva A.M., Bibik E.E., Eremkina A.K., Elfimova A.R., Salimkhanov R.K., Kovaleva E.V., Maganeva I.S., Mokrysheva N.G. (2023). Cardiovascular and metabolic status in patients with primary hyperparathyroidism: A single-center experience. Front. Endocrinol..

[32] Iglesias P., Arias J., López G., Romero I., Díez J.J. (2023). Primary Hyperparathyroidism and Cardiovascular Disease: An Association Study Using Clinical Natural Language Processing Systems and Big Data Analytics. J. Clin. Med..

[33] Maldar A.N., Shah N.F., Chauhan P.H., Lala M., Kirtane M.V., Chadha M. (2023). Differences in the Presentation and Outcome between Premenopausal and Postmenopausal Primary Hyperparathyroidism Indian Women: A Single-Center Experience. J. Mid-Life Health.

[34] Şengül Ayçiçek G., Aydoğan B.İ., Şahin M., Emral R., Erdoğan M.F., Güllü S., Başkal N., Çorapçıoğlu D. (2023). The impact of vitamin D deficiency on clinical, biochemical and metabolic parameters in primary hyperparathyroidism. Endocrinol. Diabetes Nutr. (Engl. Ed.).

[35] Soto-Pedre E., Lin Y.Y., Soto-Hernaez J., Newey P.J., Leese G.P. (2023). Morbidity Associated with Primary Hyperparathyroidism-A Population-based Study with a Subanalysis on Vitamin D. J. Clin. Endocrinol. Metab..

[36] Al-Jehani A., Al-Ahmed F., Nguyen-Thi P.L., Bihain F., Nomine-Criqui C., Demarquet L., Guerci B., Ziegler O., Brunaud L. (2022). Insulin resistance is more severe in patients with primary hyperparathyroidism. Surgery.

[37] Al-Saleh Y., AlSohaim A., AlAmoudi R., AlQarni A., Alenezi R., Mahdi L., Alzanbaqi H., Nawar S.M., AlHarbi H., ALMulla A. (2022). Primary hyperparathyroidism in Saudi Arabia revisited: A multi-centre observational study. BMC Endocr. Disord..

[38] Frey S., Bourgade R., Le May C., Croyal M., Bigot-Corbel E., Renaud-Moreau N., Wargny M., Caillard C., Mirallié E., Cariou B. (2022). Effect of Parathyroidectomy on Metabolic Homeostasis in Primary Hyperparathyroidism. J. Clin. Med..

[39] Kumari P., Arya A.K., Pal R., Sood A., Dahiya D., Mukherjee S., Rastogi A., Bhadada S.K. (2022). Comparison of Profile of Primary Hyperparathyroidism With and Without Type 2 Diabetes Mellitus: Retrospective Analysis from the Indian Primary Hyperparathyroidism Registry. Endocr. Pract..

[40] Nikooei Noghani S., Milani N., Afkhamizadeh M., Kabiri M., Bonakdaran S., Vazifeh-Mostaan L., Asadi M., Morovatdar N., Mohebbi M. (2021). Assessment of insulin resistance in patients with primary hyperparathyroidism before and after Parathyroidectomy. Endocrinol. Diabetes Metab..

[41] Antonopoulou V., Karras S.N., Koufakis T., Yavropoulou M., Katsiki N., Gerou S., Papavramidis T., Kotsa K. (2020). Rising Glucagon-Like Peptide 1 Concentrations After Parathyroidectomy in Patients with Primary Hyperparathyroidism. J. Surg. Res..

[42] Chen L., Pei J.H., Kuang J. (2020). Moderators of the Association Between Serum Parathyroid Hormone and Metabolic Syndrome in Participants with Elevated Parathyroid Hormone: NHANES 2003–2006. Horm. Metab. Res..

[43] Karras S.N., Koufakis T., Tsekmekidou X., Antonopoulou V., Zebekakis P., Kotsa K. (2020). Increased parathyroid hormone is associated with higher fasting glucose in individuals with normocalcemic primary hyperparathyroidism and prediabetes: A pilot study. Diabetes Res. Clin. Pract..

[44] Karras S., Annweiler C., Kiortsis D., Koutelidakis I., Kotsa K. (2020). Improving Glucose Homeostasis after Parathyroidectomy for Normocalcemic Primary Hyperparathyroidism with Co-Existing Prediabetes. Nutrients.

[45] (2006). World Health Organization & International Diabetes Federation. https://iris.who.int/handle/10665/43588.

[46] American Diabetes Association (2020). 2. Classification and Diagnosis of Diabetes: Standards of Medical Care in Diabetes-2020. Diabetes Care.

[47] Matthews D.R., Hosker J.P., Rudenski A.S., Naylor B.A., Treacher D.F., Turner R.C. (1985). Homeostasis model assessment: Insulin resistance and beta-cell function from fasting plasma glucose and insulin concentrations in man. Diabetologia.

[48] Hills S.A., Balkau B., Coppack S.W., Dekker J.M., Mari A., Natali A., Walker M., Ferrannini E., Report prepared on behalf of the EGIR-RISC Study Group (2004). The EGIR-RISC STUDY (The European group for the study of insulin resistance: Relationship between insulin sensitivity cardiovascular disease risk): I. Methodology and objectives. Diabetologia.

[49] Alberti K.G., Eckel R.H., Grundy S.M., Zimmet P.Z., Cleeman J.I., Donato K.A., Fruchart J.C., James W.P., Loria C.M., Smith S.C. (2009). Harmonizing the metabolic syndrome: A joint interim statement of the International Diabetes Federation Task Force on Epidemiology and Prevention; National Heart, Lung, and Blood Institute; American Heart Association; World Heart Federation; International Atherosclerosis Society; and International Association for the Study of Obesity. Circulation.

[50] Hrebícek J., Janout V., Malincíková J., Horáková D., Cízek L. (2002). Detection of insulin resistance by simple quantitative insulin sensitivity check index QUICKI for epidemiological assessment and prevention. J. Clin. Endocrinol. Metab..

[51] Matsuda M., DeFronzo R.A. (1999). Insulin sensitivity indices obtained from oral glucose tolerance testing: Comparison with the euglycemic insulin clamp. Diabetes Care.

[52] DeBoer M.D., Gurka M.J. (2017). Clinical utility of metabolic syndrome severity scores: Considerations for practitioners. Diabetes Metab. Syndr. Obes..

[53] Anghel D., Ciobica L.M., Negru M.M., Jurcut C., Otlocan L., Coca A. (2017). Bone mineral density and vitamin D levels in patients with rheumatoid arthritis. Osteoporos. Int..

[54] Chen T., Wang Y., Hao Z., Hu Y., Li J. (2021). Parathyroid hormone and its related peptides in bone metabolism. Biochem. Pharmacol..

[55] Matikainen N., Pekkarinen T., Ryhänen E.M., Schalin-Jäntti C. (2021). Physiology of Calcium Homeostasis: An Overview. Endocrinol. Metab. Clin. N. Am..

[56] Rendina-Ruedy E., Rosen C.J. (2022). Parathyroid hormone (PTH) regulation of metabolic homeostasis: An old dog teaches us new tricks. Mol. Metab..

[57] Larsson S., Jones H.A., Göransson O., Degerman E., Holm C. (2016). Parathyroid hormone induces adipocyte lipolysis via PKA-mediated phosphorylation of hormone-sensitive lipase. Cell. Signal..

[58] Gunther C.W., Legowski P.A., Lyle R.M., Weaver C.M., McCabe L.D., McCabe G.P., Peacock M., Teegarden D. (2006). Parathyroid hormone is associated with decreased fat mass in young healthy women. Int. J. Obes..

[59] Ishimura E., Okuno S., Tsuboniwa N., Norimine K., Fukumoto S., Yamakawa K., Yamakawa T., Shoji S., Nishizawa Y., Inaba M. (2013). Significant positive association between parathyroid hormone and fat mass and lean mass in chronic hemodialysis patients. J. Clin. Endocrinol. Metab..

[60] Tay Donovan Y.K., Bilezikian J.P. (2024). Interactions between PTH and adiposity: Appetizing possibilities. J. Bone Miner. Res..

[61] Kir S., Komaba H., Garcia A.P., Economopoulos K.P., Liu W., Lanske B., Hodin R.A., Spiegelman B.M. (2016). PTH/PTHrP Receptor Mediates Cachexia in Models of Kidney Failure and Cancer. Cell Metab..

[62] Hedesan O.C., Fenzl A., Digruber A., Spirk K., Baumgartner-Parzer S., Bilban M., Kenner L., Vierhapper M., Elbe-Bürger A., Kiefer F.W. (2019). Parathyroid hormone induces a browning program in human white adipocytes. Int. J. Obes..

[63] Răcătăianu N., Leach N., Bondor C.I., Mârza S., Moga D., Valea A., Ghervan C. (2017). Thyroid disorders in obese patients. Does insulin resistance make a difference?. Arch. Endocrinol. Metab..

[64] DeMambro V.E., Tian L., Karthik V., Rosen C.J., Guntur A.R. (2023). Effects of PTH on osteoblast bioenergetics in response to glucose. Bone Rep..

[65] Valea A., Ghervan C., Morar A., Pop D.D., Carsote M., Albu S.E., Georgescu C.E., Chiorean A. (2016). Hashimoto’s thyroiditis and breast cancer: Coincidence or correlation?. Arch. Balk. Med. Union.

[66] Chang E., Donkin S.S., Teegarden D. (2009). Parathyroid hormone suppresses insulin signaling in adipocytes. Mol. Cell. Endocrinol..

[67] Saxe A.W., Gibson G., Gingerich R.L., Levy J. (1995). Parathyroid hormone decreases in vivo insulin effect on glucose utilization. Calcif. Tissue Int..

[68] He X., Liu M., Ding X., Bian N., Wang J., Wang G., Liu J. (2024). Parathyroid Hormone is Negatively Correlated with Glycated Hemoglobin in Newly Diagnosed Type 2 Diabetic Patients. Int. J. Endocrinol..

[69] Tammineni E.R., Kraeva N., Figueroa L., Manno C., Ibarra C.A., Klip A., Riazi S., Rios E. (2020). Intracellular calcium leak lowers glucose storage in human muscle, promoting hyperglycemia and diabetes. eLife.

[70] Valea A., Carsote M., Moldovan C., Georgescu C. (2018). Chronic autoimmune thyroiditis and obesity. Arch. Balk. Med. Union.

[71] Weiser A., Feige J.N., De Marchi U. (2021). Mitochondrial Calcium Signaling in Pancreatic β-Cell. Int. J. Mol. Sci..

[72] Wiederkehr A., Szanda G., Akhmedov D., Mataki C., Heizmann C.W., Schoonjans K., Pozzan T., Spät A., Wollheim C.B. (2011). Mitochondrial matrix calcium is an activating signal for hormone secretion. Cell Metab..

[73] Tarasov A.I., Semplici F., Ravier M.A., Bellomo E.A., Pullen T.J., Gilon P., Sekler I., Rizzuto R., Rutter G.A. (2012). The mitochondrial Ca2+ uniporter MCU is essential for glucose-induced ATP increases in pancreatic β-cells. PLoS ONE.

[74] Misgar R.A., Bhat M.H., Rather T.A., Masoodi S.R., Wani A.I., Bashir M.I., Wani M.A., Malik A.A. (2020). Primary hyperparathyroidism and pancreatitis. J. Endocrinol. Investig..

[75] Karim M.M., Raza H., Parkash O. (2024). Recurrent acute pancreatitis as an initial presentation of primary hyperparathyroidism: A case report. World J. Clin. Cases.

[76] Carsote M., Valea A., Dumitru N., Terzea D., Petrova E., Albu S., Buruiana A., Ghemigian A. (2016). Metastases in daily endocrine practice. Arch. Balk. Med. Union.

[77] Cipriani C., Colangelo L., Santori R., Renella M., Mastrantonio M., Minisola S., Pepe J. (2020). The Interplay Between Bone and Glucose Metabolism. Front. Endocrinol..

[78] Nica S., Sionel R., Maciuca R., Csutak O., Ciobica M.L., Nica M.I., Chelu I., Radu I., Toma M. (2025). Gender-Dependent Associations Between Digit Ratio and Genetic Polymorphisms, BMI, and Reproductive Factors. Rom. J. Mil. Med..

[79] Ducy P. (2020). Bone Regulation of Insulin Secretion and Glucose Homeostasis. Endocrinology.

[80] Lee N.K., Sowa H., Hinoi E., Ferron M., Ahn J.D., Confavreux C., Dacquin R., Mee P.J., McKee M.D., Jung D.Y. (2007). Endocrine regulation of energy metabolism by the skeleton. Cell.

[81] Paracha N., Mastrokostas P., Kello E., Gedailovich Y., Segall D., Rizzo A., Mitelberg L., Hassan N., Dowd T.L. (2024). Osteocalcin improves glucose tolerance, insulin sensitivity and secretion in older male mice. Bone.

[82] Nowicki J.K., Jakubowska-Pietkiewicz E. (2024). Osteocalcin: Beyond Bones. Endocrinol. Metab..

[83] Conte C., Epstein S., Napoli N. (2018). Insulin resistance and bone: A biological partnership. Acta Diabetol..

[84] Wei J., Ferron M., Clarke C.J., Hannun Y.A., Jiang H., Blaner W.S., Karsenty G. (2014). Bone-specific insulin resistance disrupts whole-body glucose homeostasis via decreased osteocalcin activation. J. Clin. Investig..

[85] Kalra S., Joshi A., Kapoor N. (2022). Osteoporosis and diabetes: The dual pandemics. J. Pak. Med. Assoc..

[86] Mesinovic J., Fyfe J.J., Talevski J., Wheeler M.J., Leung G.K.W., George E.S., Hunegnaw M.T., Glavas C., Jansons P., Daly R.M. (2023). Type 2 Diabetes Mellitus and Sarcopenia as Comorbid Chronic Diseases in Older Adults: Established and Emerging Treatments and Therapies. Diabetes Metab. J..

[87] Popa F.L., Diaconu C., Canciu A., Ciortea V.M., Iliescu M.G., Stanciu M. (2022). Medical management and rehabilitation in posttraumatic common peroneal nerve palsy. Balneo PRM Res. J..

[88] Liu X., Chen F., Liu L., Zhang Q. (2023). Prevalence of osteoporosis in patients with diabetes mellitus: A systematic review and meta-analysis of observational studies. BMC Endocr. Disord..

[89] Al-Hariri M. (2016). Sweet Bones: The Pathogenesis of Bone Alteration in Diabetes. J. Diabetes Res..

[90] Kupai K., Kang H.L., Pósa A., Csonka Á., Várkonyi T., Valkusz Z. (2024). Bone Loss in Diabetes Mellitus: Diaporosis. Int. J. Mol. Sci..

[91] Sheu A., White C.P., Center J.R. (2024). Bone metabolism in diabetes: A clinician’s guide to understanding the bone-glucose interplay. Diabetologia.

[92] Brandt I.A.G., Starup-Linde J., Andersen S.S., Viggers R. (2024). Diagnosing Osteoporosis in Diabetes-A Systematic Review on BMD and Fractures. Curr. Osteoporos. Rep..

[93] Chuang T.L., Chuang M.H., Wang Y.F., Koo M. (2022). Comparison of Trabecular Bone Score-Adjusted Fracture Risk Assessment (TBS-FRAX) and FRAX Tools for Identification of High Fracture Risk among Taiwanese Adults Aged 50 to 90 Years with or without Prediabetes and Diabetes. Medicina.

[94] Palermo A., Tabacco G., Makras P., Zavatta G., Trimboli P., Castellano E., Yavropoulou M.P., Naciu A.M., Anastasilakis A.D. (2024). Primary hyperparathyroidism: From guidelines to outpatient clinic. Rev. Endocr. Metab. Disord..

[95] Weber T., Dotzenrath C., Dralle H., Niederle B., Riss P., Holzer K., Kußmann J., Trupka A., Negele T., Kaderli R. (2021). Management of primary and renal hyperparathyroidism: Guidelines from the German Association of Endocrine Surgeons (CAEK). Langenbecks Arch. Surg..

[96] Khan A.A., Hanley D.A., Rizzoli R., Bollerslev J., Young J.E., Rejnmark L., Thakker R., D’Amour P., Paul T., Van Uum S. (2017). Primary hyperparathyroidism: Review and recommendations on evaluation, diagnosis, and management. A Canadian and international consensus. Osteoporos. Int..

[97] Stefanova D., Ullmann T.M., Limberg J., Moore M., Beninato T., Zarnegar R., Fahey T.J., Finnerty B.M. (2020). Risk Factors for Prolonged Length of Stay and Readmission After Parathyroidectomy for Renal Secondary Hyperparathyroidism. World J. Surg..

[98] Zhao H., Zhang M., Zhen Y., Tang Y. (2022). The Relationships Between Glycated Hemoglobin and Bone Turnover Markers in Patients with Type 2 Diabetes but No Diabetic Nephropathy. Int. J. Gen. Med..

[99] Jain N., Reilly R.F. (2017). Hungry bone syndrome. Curr. Opin. Nephrol. Hypertens.

[100] Kritmetapak K., Kongpetch S., Chotmongkol W., Raruenrom Y., Sangkhamanon S., Pongchaiyakul C. (2020). Incidence of and risk factors for post-parathyroidectomy hungry bone syndrome in patients with secondary hyperparathyroidism. Ren. Fail..

[101] Ho L.Y., Wong P.N., Sin H.K., Wong Y.Y., Lo K.C., Chan S.F., Lo M.W., Lo K.Y., Mak S.K., Wong A.K. (2017). Risk factors and clinical course of hungry bone syndrome after total parathyroidectomy in dialysis patients with secondary hyperparathyroidism. BMC Nephrol..

[102] Witteveen J.E., van Thiel S., Romijn J.A., Hamdy N.A. (2013). Hungry bone syndrome: Still a challenge in the post-operative management of primary hyperparathyroidism: A systematic review of the literature. Eur. J. Endocrinol..

[103] Zavatta G., Clarke B.L. (2021). Normocalcemic Primary Hyperparathyroidism: Need for a Standardized Clinical Approach. Endocrinol. Metab..

[104] Dawood N.B., Yan K.L., Shieh A., Livhits M.J., Yeh M.W., Leung A.M. (2020). Normocalcaemic primary hyperparathyroidism: An update on diagnostic and management challenges. Clin. Endocrinol..

